# Mutant huntingtin messenger RNA forms neuronal nuclear clusters in rodent and human brains

**DOI:** 10.1093/braincomms/fcac248

**Published:** 2022-10-13

**Authors:** Socheata Ly, Marie-Cécile Didiot, Chantal M Ferguson, Andrew H Coles, Rachael Miller, Kathryn Chase, Dimas Echeverria, Feng Wang, Ghazaleh Sadri-Vakili, Neil Aronin, Anastasia Khvorova

**Affiliations:** RNA Therapeutics Institute, University of Massachusetts Chan Medical School, Worcester, MA 01655, USA; RNA Therapeutics Institute, University of Massachusetts Chan Medical School, Worcester, MA 01655, USA; RNA Therapeutics Institute, University of Massachusetts Chan Medical School, Worcester, MA 01655, USA; RNA Therapeutics Institute, University of Massachusetts Chan Medical School, Worcester, MA 01655, USA; RNA Therapeutics Institute, University of Massachusetts Chan Medical School, Worcester, MA 01655, USA; RNA Therapeutics Institute, University of Massachusetts Chan Medical School, Worcester, MA 01655, USA; RNA Therapeutics Institute, University of Massachusetts Chan Medical School, Worcester, MA 01655, USA; RNA Therapeutics Institute, University of Massachusetts Chan Medical School, Worcester, MA 01655, USA; Mass General Institute for Neurodegenerative Disease, Massachusetts General Hospital, Boston, MA 02114, USA; RNA Therapeutics Institute, University of Massachusetts Chan Medical School, Worcester, MA 01655, USA; Department of Medicine, University of Massachusetts Chan Medical School, Worcester, MA 01655, USA; RNA Therapeutics Institute, University of Massachusetts Chan Medical School, Worcester, MA 01655, USA; Program in Molecular Medicine, University of Massachusetts Chan Medical School, Worcester, MA 01655, USA

**Keywords:** Huntington’s disease, mutant *HTT* mRNA, RNA fluorescence *in situ* hybridization, nuclear RNA clusters, antisense oligonucleotides

## Abstract

Mutant messenger RNA (mRNA) and protein contribute to the clinical manifestation of many repeat-associated neurological disorders, with the presence of nuclear RNA clusters being a common pathological feature. Yet, investigations into Huntington’s disease—caused by a CAG repeat expansion in exon 1 of the *huntingtin* (*HTT*) gene—have primarily focused on toxic protein gain-of-function as the primary disease-causing feature. To date, mutant *HTT* mRNA has not been identified as an *in vivo* hallmark of Huntington’s disease. Here, we report that, in two Huntington’s disease mouse models (YAC128 and BACHD-97Q-ΔN17), mutant *HTT* mRNA is retained in the nucleus. Widespread formation of large mRNA clusters (∼0.6–5 µm^3^) occurred in 50–75% of striatal and cortical neurons. Cluster formation was independent of age and driven by expanded repeats. Clusters associate with chromosomal transcriptional sites and quantitatively co-localize with the aberrantly processed N-terminal exon 1-intron 1 mRNA isoform, *HTT1a*. *HTT1a* mRNA clusters are observed in a subset of neurons from human Huntington’s disease post-mortem brain and are likely caused by somatic expansion of repeats. In YAC128 mice, clusters, but not individual *HTT* mRNA, are resistant to antisense oligonucleotide treatment. Our findings identify mutant *HTT*/*HTT1a* mRNA clustering as an early, robust molecular signature of Huntington’s disease, providing *in vivo* evidence that Huntington’s disease is a repeat expansion disease with mRNA involvement.

## Teaser

Using Huntington’s disease mouse models and patient brains, Ly *et al.* identify the widespread formation of nuclear mutant HTT messenger RNA clusters. These clusters are resistant to antisense treatment, were observed in most neurons as early as 1 month and are co-localized with an aberrantly spliced short exon 1-containing isoform HTT1a.

## Graphical Abstract

In wild-type neurons, HTT mRNA does not cluster and HTT is not expressed. In Huntington's disease neurons, mutant HTT and HTT form clusters at HTT gene loci.

**Graphical abstract:**
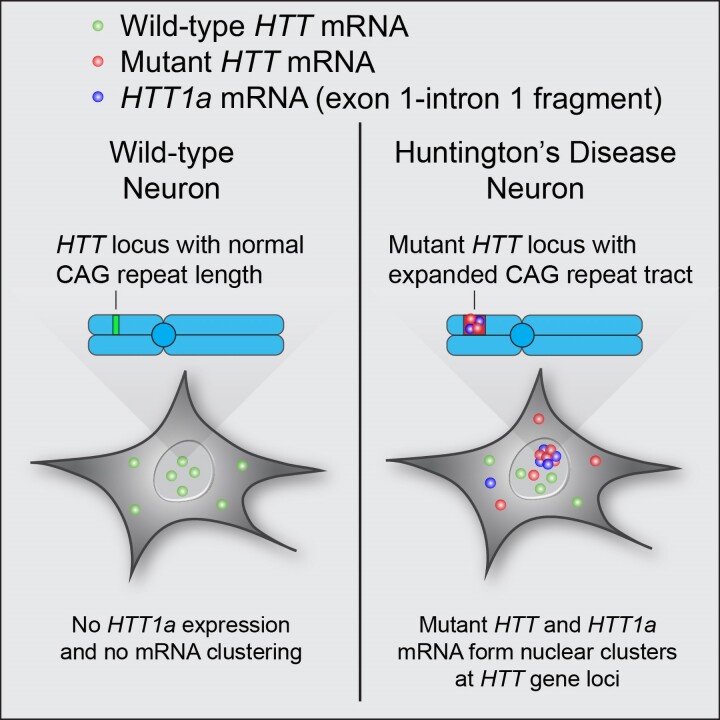

## Introduction

Inherited autosomal dominant neurological and neuromuscular diseases, including Huntington’s disease (HD), myotonic dystrophy, familial amyotrophic lateral sclerosis (ALS) and frontotemporal dementia (FTD), are characterized by a microsatellite expansion of nucleotide repeats. Characteristic hallmarks of myotonic dystrophy, FTD and ALS include nuclear accumulation of mutated transcripts and formation of aberrant nuclear messenger RNA (mRNA) inclusions,^1^ likely facilitated by long repeat-based hairpins.^2,3^ For HD, these characteristic hallmarks have not yet been identified *in vivo*.

HD is caused by a CAG repeat expansion in exon 1 of the *huntingtin* (*HTT*) gene,^4^ resulting in transcription of CAG repeat-expanded mutant *HTT* mRNA and translation of polyglutamine (poly Q) repeat-expanded mutant HTT protein. Although wild-type and mutant HTT are first expressed ubiquitously at the embryonic stage,^5–7^ clinical symptoms (motor and psychiatric) develop later, typically during midlife. Age of clinical onset is inversely correlated to repeat expansion length: ∼39 to mid-40s repeats cause adult-onset HD,^8^ while >60 repeats cause juvenile HD.^9^ Clinical symptoms are primarily driven by neuronal loss in the striatum and cortex.^10,11^ However, the molecular mechanisms underlying the link between repeat expansion and disease progression are unclear.

The role of mutant HTT protein has been the primary subject of investigations into HD progression, with a variety of perturbed cellular functions being reported. These functions include aggregation,^12^ production of aberrantly processed toxic N-terminal isoforms,^13–16^ disruption of nuclear integrity,^17^ clogging of nucleocytoplasmic transport^18^ and interference with synaptic connectivity and survival in striatal projections.^19^ Recent GWAS studies, however, show that the length of uninterrupted CAG repeats—not polyQ tract—defines the age of onset,^20,21^ highlighting an underappreciated role of mutant DNA/RNA in HD. Furthermore, GWAS studies identified mismatch repair genes as disease modifiers. Mismatch repair is the primary mechanism behind the somatic expansion of nucleotide repeats, a well-described phenomenon in HD.^22–24^ Although the clinical significance of expanded CAG repeats has been shown, it is unclear how mutant mRNA contributes to HD.

Multiple lines of evidence indicate that CAG repeats can form extensive secondary structures.^2^ Repeat-containing RNAs create a framework for multivalent interactions, resulting in repeat length-driven phase separation and clustering that has been observed *in vitro* (with purified RNA).^25^ In cells, artificial overexpression of expanded CAG, but not CAA, repeat-containing *HTT* exon 1 fusions causes changes in RNA intracellular localization with some nuclear clustering (detected by a repeat-targeting probe).^26,27^ In a native genomic context, the pattern of *HTT* mRNA expression is affected by cellular origin, with neurons displaying preferential (≥60%) nuclear localization.^28^ A deeper investigation into the effect of CAG expansion on *mutant HTT* (*mHTT*) mRNA intracellular localization in the brain is warranted.

Using branched fluorescence *in situ* hybridization (FISH) technology,^28,29^ we investigated the impact of repeat expansion on *mHTT* subcellular localization in two HD transgenic models, YAC128 and BACHD-97Q-ΔN17, and post-mortem brain sections from an HD patient. In HD mouse brains, we observed a repeat-driven increase in nuclear *mHTT* mRNA retention and widespread (≥80% of neurons) formation of nuclear mRNA clusters (∼0.6–5 µm3). Nuclear clusters were highly specific to neuronal *mHTT* mRNA, localized to *HTT* transcriptional sites and resistant to treatment with antisense oligonucleotides (ASOs). Nuclear cluster prevalence was independent of age. We further demonstrate close to complete cluster co-localization with the aberrantly processed N-terminal exon 1-intron 1 transcript *HTT1a*.^13^*HTT1a* overexpression is likely to nucleate transcription site clustering. In the human HD brain, nuclear *HTT1a* clusters were detectable, reinforcing their clinical relevance. Collectively, our findings reveal an early, robust molecular feature of HD neuropathology strongly linked to CAG repeat expansion in mRNA that might be resistant to clinically-advanced treatments currently under evaluation.

## Materials and methods

### Contact for reagent and resource sharing

Further information and requests for resources and reagents should be directed to and will be fulfilled by the Lead Contact, Anastasia Khvorova (Anastasia.Khvorova@umassmed.edu).

### Experimental model and subject and details

#### Mice and ethics statements

Wild-type FVB female mice, and B97-ΔN17, B31-ΔN17^30^ and YAC128^31^ heterozygous FVB male mice were obtained from The Jackson Laboratory (Supplementary Table 1). All animals have been maintained in a maximum of five a cage in a specific pathogen-free facility under standard conditions with access to food and water *ad libitum* at the University of Massachusetts Chan Medical School (UMass Chan). B97-ΔN17, B31-ΔN17 and YAC128 male mice were bred with wild-type female mice, resulting in mixed wild-type and heterozygous litters weaned from their mothers between 18 and 21 days of age. Genotyping was performed by PCR using DNA extracted from ear punches taken at the time of wean. For each experiment, mice of mixed sex were randomized into experimental groups. No sex-based differences were observed. For YAC128 mice, a previous study reported no significant differences between male and female mice.^32^ Furthermore, all experimental groups contained *n* = 3 mice which precluded us from performing any statistical analysis based on sex. All procedures were completed in accordance with the National Institutes of Health Guideline for Laboratory Animals and were approved by the UMass Chan IACUC (Protocol #A2411).

#### Mouse tissue collection and sectioning

Mice were euthanized according to our institutional IACUC protocol (#A2411). At each time point, mice were deeply anesthetized with tribromoethanol and perfused intracardially with 20 ml 1X PBS buffer. Tissues were dissected out and placed (brain eye bulbs facing upwards) in disposable cryomold (Polysciences Inc. #18986–1), and frozen in O.C.T. embedding medium (Tissue-Tek #4583) in a dry ice/methanol bath. Brains were stored at −80°C until use and transferred overnight at −20°C prior to sectioning. Brains were sliced into 20 μm brain sections using a cryostat (temperatures: sample holder −13°C, blade −12°C) (ThermoFisher CryoStar™ NX70) and mounted on superfrost slides (Fisher #1255015). Slides were stored at −80°C until further experiments.

#### Human primary cells and brain samples

Human tissue was kindly provided by the Sandri-Vakili lab (MassGeneral Institute for Neurodegenerative Disease) as well as from the NIH NeuroBioBank (University of Pittsburgh Brain Tissue Donation Program and University of Maryland). We do not have access to the master list to re-identify subjects. This activity is not considered to meet federal definitions under the jurisdiction of an institutional review board, and thus, is exempt from the definition of human subject.

### Method details

#### Oligonucleotides

Sequences and chemical modification patterns of the ASOs are described in Supplementary Table 2. LNA GapmeR ASOs, designed by IONIS Pharmaceuticals,^33^ were purchased from Exiqon.

#### Animal stereotaxic injections of oligonucleotides

All mice used were adult FVB/NJ YAC128, 12 weeks old at the time of the injection. Prior to injection, mice were deeply anesthetized with 1.2% Avertin (Sigma #T48402). Four nanomoles ASO*^NTC^* or ASO*^HTT^* (*n* = 3 mice per treatment group), diluted at 2 nmol/µl in 25 mM Mg buffer, were administered by direct bolus microinjection into the right striatum by stereotaxic placement; coordinates (relative to bregma) were +1.0 mm anterio-posterior, + 2.0 mm medio-lateral and +3.0 mm dorso-ventral. All injection surgeries were performed using sterile surgical techniques and were accomplished using a standard rodent stereotaxic instrument and an automated microinjection syringe pump (Digital Mouse Stereotaxic Frame; World Precision Instrument #504926). No adverse events were observed. Mice were euthanized 4 weeks post-injection and brains were harvested.

#### Fluorescent *in situ* hybridization

FISH enables single-cell detection of transcripts *in situ*, and accurate quantification of the relative levels of mRNA expression. B97-ΔN17, B31-ΔN17 and YAC128 mouse models express both wild-type *Mm Htt* mRNA and mutant *Hs HTT* mRNA. We compared the expression level of *Hs HTT* mRNA with the expression level of *Mm Htt* mRNA and housekeeping *Hprt* mRNA. See Supplementary Table 3 for the list of probes used.

#### Sample preparation

Mouse brain sections obtained on a cryostat were prepared as described by the manufacturer protocol for fresh frozen tissue (ACDBio #320513). Briefly, sections were fixed in 10% formalin for 15–20 min at 4°C and washed three times in PBS. Sections were dehydrated by sequential incubation in 50, 70 and 100% ethanol for 5 min at room temperature and air dried for 5 min at room temperature. During this time, the hydrophobic barrier around the sections can be drawn. Sections were incubated for 20–30 min in protease solution (Pre-treatment IV) at room temperature. Sections were washed twice in PBS and processed for FISH.

FISH was performed using the RNAscope® Fluorescent Multiplex kit (ACDBio #320850) following the manufacturer’s instruction (ACDBio #320293). Prior to any experiment, we ensured that the probes were pre-warmed at 40°C and cooled to room temperature to dissolve any crystal formed in the probe solution during storage at 4°C. Following sample preparation, samples were incubated with the target probe in the HybEZ^™^ oven at 40°C for 3 h. The signal was amplified by incubation with the pre-amp, amp and label probes for 30 min each at 40°C. Between each incubation, samples were incubated in a wash buffer twice for 2 min at room temperature. Following signal amplification, sample nuclei were stained with DAPI solution for 1 min, mounted in ProLong™ Gold antifade medium (ThermoFisher #P36930) and dried at room temperature overnight.

#### FISH and immunofluorescence

Detection of SC35 by immunofluorescence (IF) was performed following FISH. Briefly, the FISH procedure was performed as previously described by the manufacturer protocol followed directly by IF. Brain sections were incubated for 1 h in blocking solution (2% Normal goat serum, 0.01% Triton-X in PBS) at room temperature. Slides were washed three times for 5 min in PBS. Brain sections were incubated in primary antibodies diluted in PBS overnight at room temperature. Slides were washed three times for 5 min in PBS and incubated for 1 h at room temperature in secondary antibodies diluted in PBS. Slides were washed three times for 5 min in PBS, mounted in ProLong™ Gold antifade medium and dried at room temperature overnight.

#### Chromogenic ISH assay for human brains

Due to excess autofluorescence caused by the presence of lipofuscin in aged human brains, we could not use the RNAscope FISH assay as described earlier. Instead, we used the chromogenic RNAscope 2.5 HD Duplex Assay (ACDBio #322430) according to the manufacturer’s protocol for fresh frozen brains (ACDBio #320536-TN). Brain sections (20 µm thick) were cryosectioned and placed on Superfrost slides and stored at −80°C until ready to use. Samples were fixed by immersing the slides in pre-chilled 10% neutral-buffered formalin and incubated at 4°C for 1 h. Slides were dehydrated by immersion in increasing concentrations of ethanol for 5 min at room temperature: 70% ethanol, 100% ethanol and 100% ethanol again. Slides were briefly dried at room temperature for 5 min and then hydrophobic barriers were drawn using an Immedge pen. Four drops of hydrogen peroxide were added to each sample, followed by 10 min incubation at room temperature, and then rinsed with PBS. Four drops of Protease IV solution were added to each sample, followed by 30 min incubation at room temperature, and then five PBS washes.

C1 and C2 RNAscope probes were hybridized for 2 h at 40°C in the HybEZ Oven followed by overnight incubation in 5 × SSC buffer at room temperature. The next day, the slides were washed twice with wash buffer. Four drops of Amp 1 were added to each slide and incubated for 30 min at 40°C followed by two washes with wash buffer. This process was repeated with Amp 2 (15 min at 40°C), Amp 3 (30 min at 40°C), Amp 4 (15 min at 40°C), Amp 5 (30 min at room temperature), Amp 6 (15 min at room temperature), Red solution (10 min at room temperature), Amp 7 (15 min at 40°C), Amp 8 (30 min at 40°C), Amp 9 (30 min at room temperature), Amp 10 (15 min at room temperature) and Green solution (10 min at room temperature). The slides were then counterstained in 50% hematoxylin staining solution for 30 s at room temperature, immediately washed with tap water, rinsed with 0.02% ammonia water and then washed with tap water five times. The slides were then baked in the HybEZ oven at 60°C for 1 h, mounted with VectaMount Permanent Mounting Medium (H-5000), covered with 24 × 50 mm cover glass, and air dried overnight. Images were then acquired on a Leica DMi8 brightfield microscope.

#### Microscopy

Two confocal microscopes were used to acquire images. First, images were acquired with a CSU10B Spinning Disk Confocal System scan head (Solamere Technology Group) mounted on a TE-200E2 inverted microscope (Nikon) with a 100x Plan APO oil-immersion objective and a Coolsnap HQ2 camera (Roper Technologies). 16-bit image stacks were acquired using Micro-Manager v1.4.19 by imaging 15 µm z-stacks (step size = 0.5 µm) through the tissue sections. Field of view dimensions were approximately 33.89 µm^2^ (512 pixels^2^) with voxel size 0.0662 × 0.0662 × 0.5 µm^3^. Image acquisition order was as follows: x, y, channel, z. Image acquisition settings were kept consistent for all experiments and empirically determined using the manufacturer’s 3plex positive control probeset (ACDBio #320881) and 3plex negative control probeset (ACDBio #320871): 350 nm laser, 200 ms integration; 488 nm laser, 500 ms; 543 nm laser, 500 ms; gain 500 for all channels. Images were processed using ImageJ v1.53c.^34,35^ The percentage of saturated pixels in each image was less than 0.01%, limited only to the centroids of clusters, limiting potential bias in the volumetric calculations.

All image quantification was performed using images acquired from this microscope.

Second, a Leica SP8 LIGHTNING laser scanning confocal microscope equipped with an HC PL APO CS2 63x/1.40 OIL objective and Diode 405, OPSL 488, OPSL 552, Diode 638 laser lines were used to acquire images on the manufacturer’s LAS X 3.5.5.19976 software. A PMT detector was used for the 405 channel and HyD detectors were used for all other channels. Each channel was acquired sequentially with excitation lasers (at 1% intensity for all channels) and emission ranges as follows (all in nm): 405 (410–488), 488 (493–719), 552 (559–629), 638 (658–789). Zoom (ranging from 1 to 2.5) and voxel size (ranging from xy = 40 to 50 nm; z: 300 to 500 nm) varied between images. Samples were mounted with ProLong Glass (RI = 1.518) and Leica Immersion Oil (RI = 1.51) was used. Images were processed using the LIGHTNING deconvolution package with default settings for Prolong Glass mounting media. All images acquired on this microscope were used for qualitative assessment and never quantitatively analysed.

### Quantification and statistical analysis

#### Statistics

No statistical methods were performed to pre-determine sample sizes. Data analyses were performed using GraphPad Prism 8.4.3 software (GraphPad Software, LLC). The D’Agostino-Pearson omnibus normality test was used on the raw data and residuals to test for normality, and confirmed that nuclear fraction data and foci volume data are not normally distributed. For each sample group, at least 100 individual cells were analysed. The statistical test used is also reported in the figure legends as well as the level of statistical significance, which is denoted by asterisks (^∗^, *P* < 0.05; ^∗∗^, *P* < 0.01; ^∗∗∗^, *P* < 0.001; ^∗∗∗∗^, *P* < 0.0001).

#### Image processing

Image processing of RNAscope images was performed with ImageJ (v1.53c) using a macro written in-house (accessible at https://github.com/socheataly/imagej) (Supplementary Fig. 1). First, nuclei were segmented by convolving the Hoechst 33342 or DAPI signal with a Gaussian blur (σ = 10 pixels), ‘Default’ thresholding algorithm (stack histogram enabled) to generate a binary mask of the nucleus in 3D. RNAscope puncta detection was performed by first using a difference of Gaussians filter (*σ*_1_ = 2 pixels, *σ*_2_ = 4 pixels) followed by thresholding using the Otsu algorithm (stack histogram enabled)^36^ or a manual threshold (250 intensity), whichever value was smaller. Setting a minimum threshold value was necessary to correctly threshold images with no RNAscope foci. Next, the 3D Objects Counter plugin^37^ was used to define these puncta as 3D objects and quantify their volumes (minimum size = 25 voxels). RNAscope foci were assigned to the nucleus if it overlapped with the nuclear mask; otherwise, the foci were assigned to the cytoplasm. Nuclear fraction was calculated in individual cells using the following equation: (nuclear foci)/(nuclear foci + cytoplasmic foci). As previously described, the total number of objects varies widely from cell to cell.^28^

Clusters were defined as RNAscope puncta with volumes of at least 0.6 µm^3^ (corresponding to 274 voxels), which is approximately four times the median volume of typical RNAscope foci as measured on our system. Parameters were kept consistent between all sample groups and were determined by using the manufacturer’s 3plex positive control and 3plex negative control probesets such that no processed RNAscope signal was detectable in the 3plex negative control sample (see ‘Microscopy’ section).

To quantify co-localization, RNAscope images were processed as described above and then separate channels were compared using the “Image Calculator > ‘AND’” function in ImageJ v1.53c. The resulting image was processed with the 3D Objects Counter Plugin and foci were considered to be co-localized if the resulting objects were at least 25 voxels in volume, which is the same cut-off used to detect RNAscope foci.

## Results

### Experimental system for multiplex evaluation of *huntingtin* mRNA localization *in vivo*

**Figure 1:**
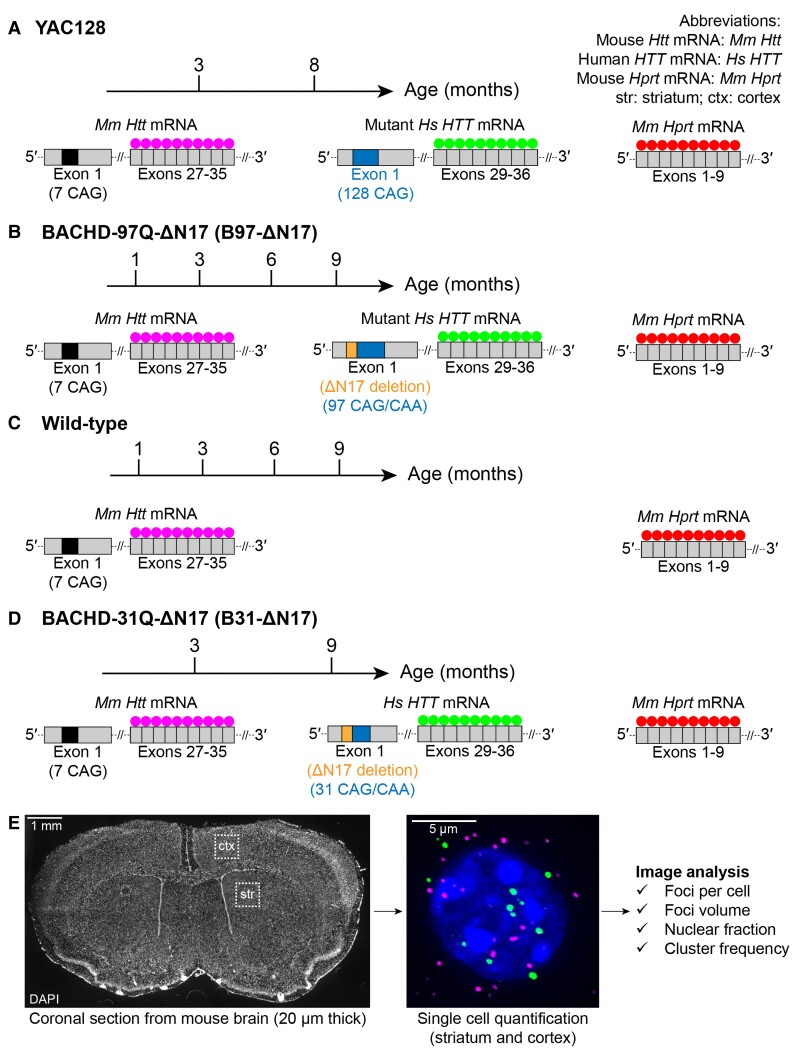
**Experimental system for multiplex evaluation of wild-type *Htt* and *Hs HTT* mRNA subcellular localization in brain slices.** (**A–D**) Dual-colour FISH probe sets targeting: mouse (*Mm*) *Htt* mRNA in exons 27–35, human (*Hs*) *HTT* mRNA in exons 29–36 and *Mm Hprt* mRNA in exons 1–9 in (**A**) YAC128, (**B**) B97-ΔN17, (**C**) wild-type and (**D**) B31-ΔN17 mice. (**E**) Regions of the mouse brain used for RNAscope image analysis.

To investigate the localization of wild-type and CAG-expanded mutant mRNA in the context of the same cell, we selected YAC128^31^ and BACHD-ΔN17-97Q (referred to as B97-ΔN17)^30,38^ mice as models (Fig. 1). YAC128 carries wild-type mouse (*Mm*) *Htt* mRNA (7 CAG) and a full-length human *(Hs) HTT* transgene containing a mostly pure expanded CAG tract (82 of 128 repeats are uninterrupted CAG) (Fig. 1A). B97-ΔN17 expresses wild-type *Mm Htt* mRNA and *Hs HTT* transgene containing an expanded CAG/CAA tract (97 repeats). The first 17 amino acids of *Hs HTT* are deleted (Fig. 1B), causing increased nuclear localization of HTT protein and acceleration of HD pathology.^30^ For controls, we used wild-type mice (carrying *Mm Htt*) (Fig. 1C) and B31-ΔN17 mice carrying *Mm Htt* and the same *Hs HTT* transgene as B97-ΔN17 mice, but with 31 repeats instead of 97^30^ (Fig. 1D). To assess the effect of age on mRNA localization, three mice (male and female) were evaluated at different ages: YAC128 (3, 8 months), B97-ΔN17 (1, 3, 6, 9 months), wild-type (1, 3, 6, 9 months) and B31-ΔN17 (3, 9 months). For HD models, these time points span time before and after the presentation of behavioural phenotypes, which occur at ∼6–7 months of age.

To determine the intracellular localization of mRNA variants in the cortex and striatum, mouse brains from each group were sliced stereotactically and ∼100 cells per region were randomly selected for analysis with highly-sensitive and specific RNAscope FISH technology (Fig. 1E) .^28,29^Fig. 1 shows the design of species-specific probes targeting *Hs HTT* (green) and *Mm Htt* (magenta) mRNA. *Mm, Hprt* mRNA was used as a housekeeping control (magenta). To count the number of mRNA foci per cell and determine nuclear versus cytoplasmic distribution, we quantified RNA foci (individual transcripts) in three dimensions throughout the volume of each cell (see Methods; Supplementary Fig. 1). Background co-localization between *Mm Htt* and *Hs HTT* was minimal in nucleus and cytoplasm (Supplementary Fig. 2A and B), confirming the ability of multiplexed FISH to selectively detect wild-type and repeat-expanded mRNA at single-cell resolution.

### Repeat expansion increases nuclear retention of *Hs HTT* mRNA in cortex and striatum of HD mice

**Figure 2:**
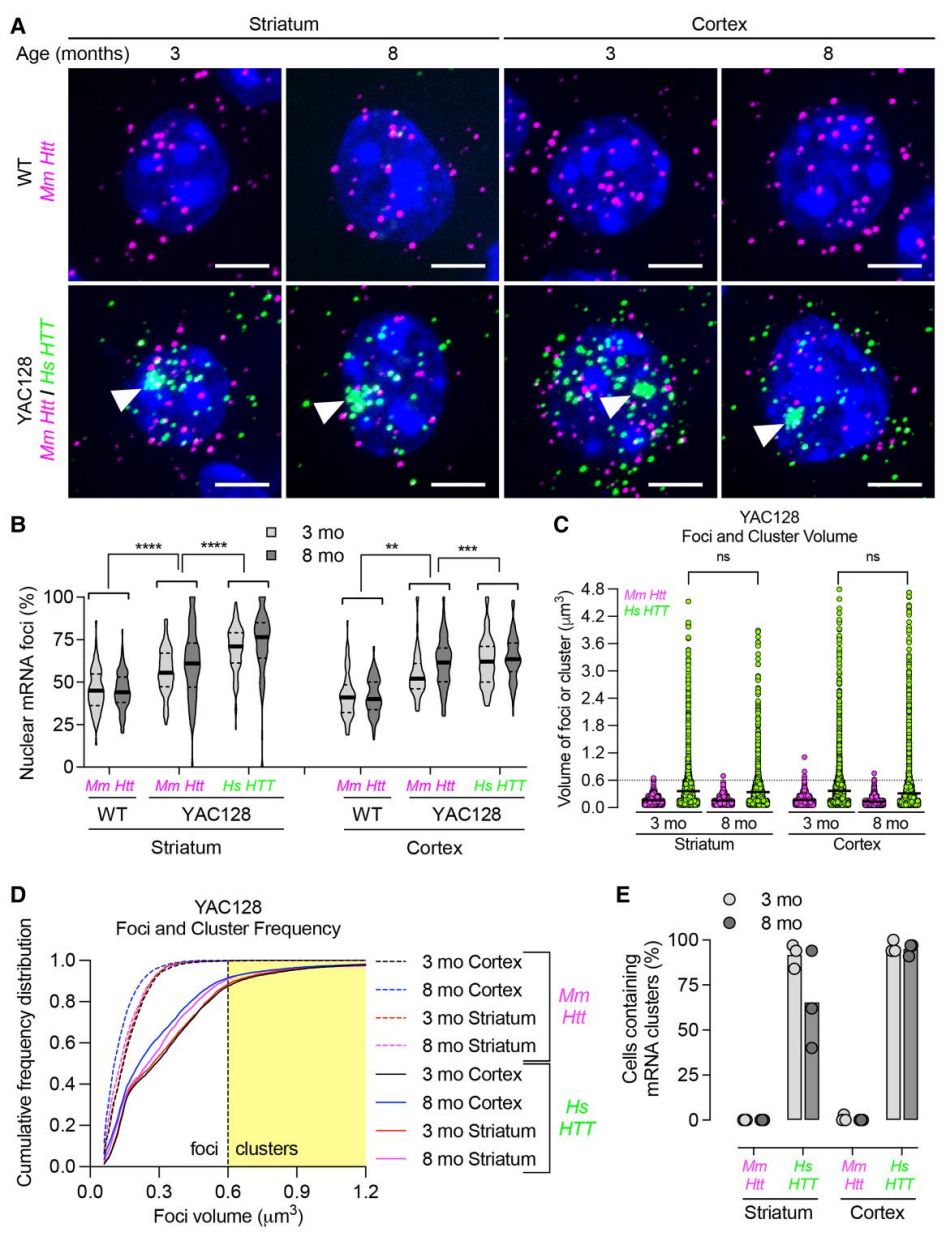
**Repeat expansion increases nuclear retention of mutant *Hs HTT* mRNA and forms clusters in YAC128 mouse striatum and cortex.** (**A**) *Mm Htt* and *Hs HTT* mRNAs were detected in YAC128 mouse striatum and cortex by FISH. Nuclei labelled with Hoechst. Scale bar, 5 µm. (**B**) Percentage of nuclear *Mm Htt* and *Hs HTT* mRNAs in wild-type (WT) and YAC128 mice at 3 and 8 months old (*n* = ∼100 cells per region pooled from three mice). (**C**) Scatter plot showing the volume of individual mRNA foci or clusters (see Methods for volume calculation). Each point represents the volume of individual mRNA foci. (**D**) Cumulative frequency distribution plot of RNA foci volume. The yellow shaded area represents the cut-off for a cluster, which is defined as ≥0.6 µm^3^. The thick line represents the mean. (**E**) Percentage of cells containing *Mm Htt* or *Hs HTT* mRNA clusters in YAC128 mouse striatum and cortex (*n* = ∼100 cells per brain region pooled from three mice, each point represents a mouse). For all panels, ns = not significant, **P < 0.01, ***P < 0.001, ****P < 0.0001, one-way ANOVA, Bonferroni’s multiple comparisons test.

Within a given animal, we observed significant cell-to-cell variability in *Mm Htt* and *Hs HTT* neuronal expression, consistent with our previous report.^28^ The number of *Htt* and *HTT* mRNA foci ranged from 0 to over 30 per cell (Supplementary Fig. 2C and D). Across ages of wild-type and HD models (except 1-month-old wild-type mice), expression patterns of *Mm Htt* mRNA stayed consistent. The median number of foci was ∼15–20 per cell with a similar nuclear versus cytoplasmic distribution: 8–10 nuclear versus 7–10 cytoplasmic foci per cell (Supplementary Fig. 2C and D). Nuclear retention of *Mm Htt* in YAC128 was slightly increased (∼50–60%) compared to wild-type mice (∼40–45%) but was still lower than that of *Hs HTT* mRNA (∼60–75%) (Fig. 2A and B), suggesting that *Hs HTT* expression minimally alters localization of *Mm Htt* mRNA.

**Figure 3:**
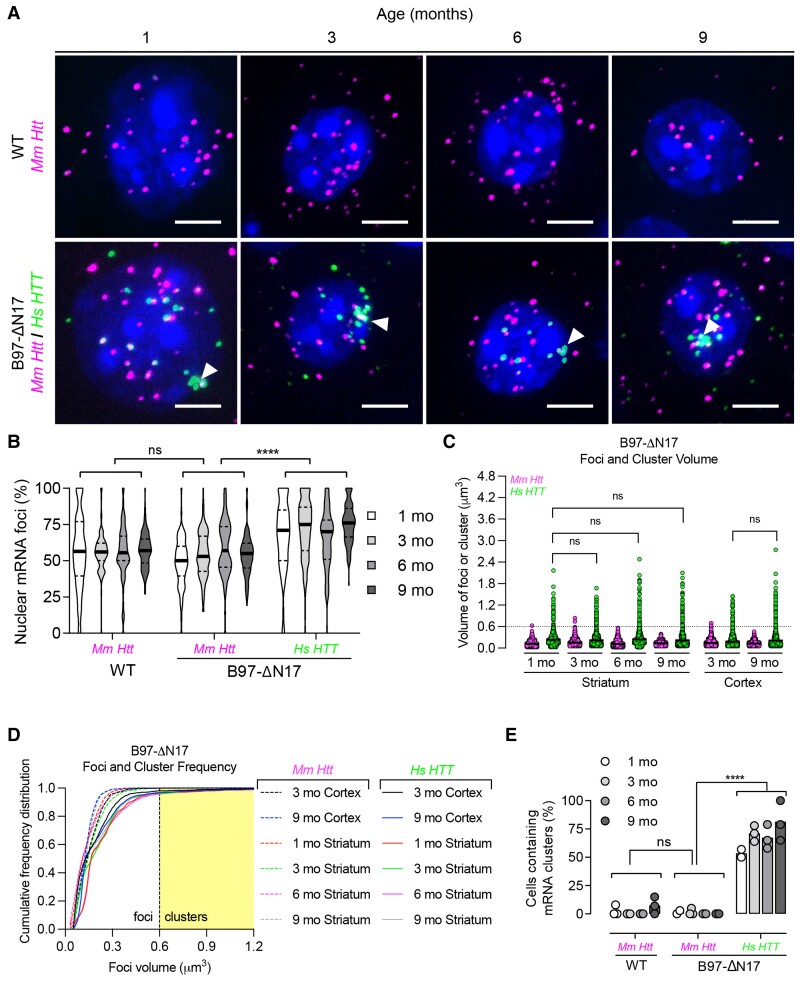
**Repeat expansion increases nuclear retention of mutant Hs HTT mRNA and forms clusters in B97-ΔN17 mouse striatum.** (**A**) *Mm Htt* and *Hs HTT* mRNAs were detected in B97-ΔN17 mouse striatum by FISH. Nuclei labelled with Hoechst. Scale bar, 5 µm. (**B**) Percentage of nuclear *Mm Htt* and *Hs HTT* mRNAs in wild-type (WT) and B97-ΔN17 mice at 1, 3, 6, and 9 months old (*n* = ∼100 cells per brain region pooled from three mice). (**C**) Scatter plot showing the volume of individual mRNA foci or clusters (see Methods for volume calculation). Each point represents the volume of individual mRNA foci. (**D**) Cumulative frequency distribution plot of RNA foci volume. The yellow shaded area represents the cut-off for a cluster, which is defined as ≥0.6 µm^3^. The thick line represents the mean. (**E**) Percentage of cells containing *Mm Htt* or *Hs HTT* mRNA clusters in B97-ΔN17 mouse striatum (*n* = ∼100 cells per brain region pooled from three mice, each point represents a mouse). For all panels, ns = not significant, **P < 0.01, ***P < 0.001, ****P < 0.0001, one-way ANOVA, Bonferroni’s multiple comparisons test.

In both HD models (all ages), we observed higher nuclear retention (∼75%) of *Hs HTT* mRNA compared to *Mm Htt* mRNA (Fig. 2A and B, Fig. 3A and B). However, single-cell resolution uncovered variations in *Hs HTT* mRNA localization patterns. In B97-ΔN17, *Hs HTT* mRNA had a significantly lower number of cytoplasmic foci compared to *Mm Htt* mRNA (median = 2–4 versus 7–11, respectively, *P* < 0.0001), while the number of nuclear foci was consistent (median of 7–10 for *Mm Htt* and *Hs HTT*) (Supplementary Fig. 2C). In YAC128 mice, there was a higher number of *Hs HTT* mRNA nuclear foci compared to *Mm Htt* (median = 19–23 versus 11–13 nuclear foci, respectively, *P* < 0.0001), while the number of cytoplasmic foci was constant (median = 10–11 for *Mm Htt* and *Hs HTT*) (Supplementary Fig. 2D). These data suggest that the mechanism underlying nuclear retention might differ between HD models.

Increased nuclear retention was specific to mutant *Hs HTT* mRNA and was not seen with the housekeeping gene *Mm Hprt*, suggesting that this observation is not a global phenomenon, but rather specific for certain mRNAs **(**Supplementary Figs 3–6**)**. These results support the hypothesis that repeats expansion leads to further nuclear enrichment of neuronal *HTT* mRNA *in vivo*, defining a molecular hallmark of HD at the RNA level.

### Widespread repeat-dependent nuclear *Hs HTT* mRNA clusters occur in HD mice

While using RNAscope to visualize *Hs HTT* mRNA in YAC128 and B97-ΔN17 mouse brain, we found that *Hs HTT* RNA clusters in a distinct population of extremely large foci (white arrowheads in Figs 2A and 3A) in the majority of striatal and cortical neurons. Randomly selected neuronal images are shown in Supplementary Fig. 7 illustrate that *Hs HTT* mRNA clustering is localized in the nucleus and easily observable. Given the importance of nuclear mRNA clusters in other repeat-driven disorders,^1,39–41^ we investigated this phenomenon further.

Using standard image processing techniques in ImageJ (Supplementary Fig. 1; see Methods),^34–37^ we calculated cluster volumes. Normal foci volume, indicative of a single mRNA transcript, was ∼0.15 µm^3^ (ranging from 0.05 to 0.3 µm^3^), whereas clusters ranged from 0.6 to ∼5 µm^3^. For automated quantification, we used a formal cut-off of 0.6 µm^3^ for clusters—a volume roughly four times that of typical foci measured in our system. In addition to volumetric differences, fluorescent intensities of individual pixels were significantly higher in clusters compared to foci: 30 000–60 000 versus 6000–25 000, respectively (16-bit images with a maximum intensity of 65 535, or 2^16^).

In three- and eight-month-old YAC128 mice, *Hs HTT* clusters up to 5 µm^3^ were detected, whereas *Mm Htt* did not form clusters (Fig. 2C). Indeed, a cumulative frequency distribution plot showed a clear shift in the volumes of *Hs HTT* RNA versus *Mm Htt* RNA (Fig. 2D). Between three- and eight-month-old mice, there was no statistically significant difference in foci or cluster volume in striatum or cortex. In the cortex, 95–96% of neurons contained *Hs HTT* clusters at both ages (Fig. 2E). In striatum, however, the frequency of *Hs HTT* clusters decreased from 92% (3 months) to 62% (8 months) (Fig. 2E). This decrease in clusters (not statistically significant) could be due to cluster-containing striatal neurons preferentially dying during disease progression.

In B97-ΔN17 mouse striatum (Fig. 3) and cortex (Supplementary Fig. 4), over 50% of neurons contained at least one *Hs HTT* cluster per nucleus as early as 1 month. This molecular event is observed at all ages (1, 3, 6, 9 months), and both frequency and size of clusters were consistent over time (Fig. 3C–E, Supplementary Fig. 4B–D). Like YAC128 mice, the appearance of RNA clusters occurs prior to the onset of overt behavioural symptoms and biochemical readouts (i.e. HD protein aggregates)^30,31^ in B97-ΔN17 mice. However, frequency and size of clusters differed between the two models. *Hs HTT* clusters in B97-ΔN17 were less frequent (54–81%) compared to YAC128 (62–96%), and smaller in size (∼2 µm^3^) compared to YAC128 (∼5 µm^3^). Since clusters potentially arise from multivalent CG base-pairing interactions, the presence of the interrupting CAA repeat in B97-ΔN17 mice could be responsible for these differences.^25^ It is also possible that differences are due to the overall repeat length itself, the 51-nucleotide deletion (17 amino acids) in *HTT* exon 1 of B97-ΔN17 mice, or the higher nuclear expression of *Hs HTT* in YAC128 (19–23 foci per cell) versus B97-ΔN17 (7–10 foci per cell).

For all cluster-positive cells in HD models, only a single cluster was observed per cell. Although automated imaging did detect a very small percentage of cells (<5% in all animals across all ages) containing cytoplasmic clusters, this was likely due to intrinsic error in nuclear segmentation analysis. Upon visual examination, all clusters were nuclear. This phenomenon was limited to neurons (data not shown), and no clusters were detected in liver or muscle of B97-ΔN17 mice (Supplementary Fig. 4F). The selective presence of clusters in neurons provides additional support to cluster involvement in HD pathology.

No clustering was observed with *Mm Htt* or *Mm Hprt* mRNA in YAC128 (Fig. 2C–E; Supplementary Fig. 3) or B97-ΔN17 **(**Fig. 3C–E, Supplementary Figs 4B–E, 5C–E) mice at all ages, indicating that this phenomenon is caused by the expanded repeat tract. To confirm that clusters are not caused by the presence of the transgene itself, we analysed B31-ΔN17 mice and did not detect *Hs HTT*, *Mm Htt*, or *Mm Hprt* clusters at appreciable levels (Supplementary Fig. 6).

### The aberrantly processed n-terminal *HTT* fragment, *Hs HTT1a*, co-clusters with *Hs HTT* RNA

**Figure 4:**
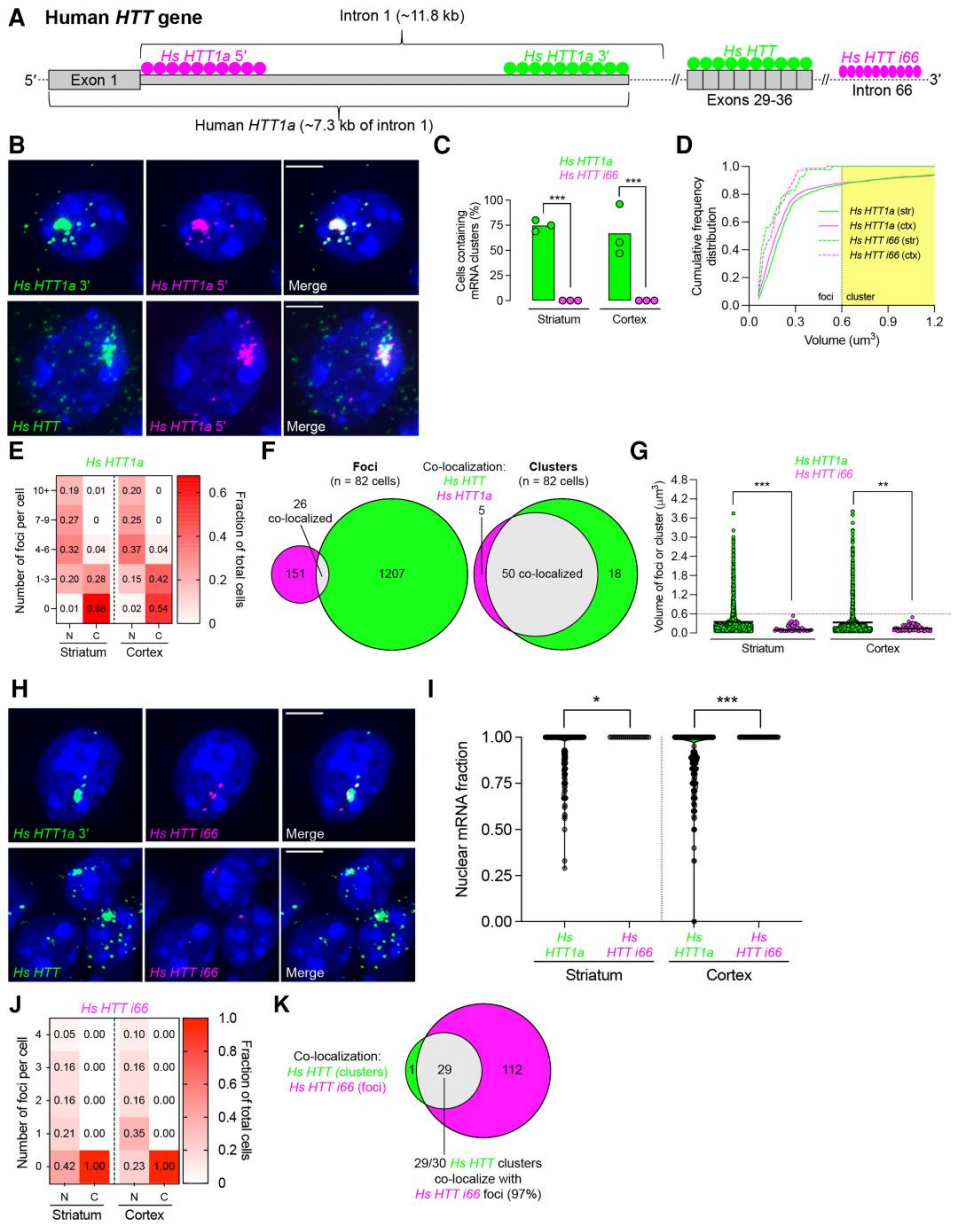
**Hs HTT1a, the aberrantly spliced exon 1-intron 1 fragment, is present in the cytoplasm and forms clusters that co-localize with Hs HTT clusters.** (**A**) Gene schematic showing *Hs HTT1a* and *Hs HTT i66*. Filled circles indicate regions where FISH probes were designed. (**B**) Confocal microscope images of YAC128 mouse striatum (3 months old) detected by FISH. DAPI. Scale bar, 5 µm. (**C**) Percentage of cells containing *Hs HTT1a* or *Hs HTT i66* mRNA clusters in YAC128 mouse striatum and cortex (*n* = ∼300 cells per brain region pooled from three mice, each point represents a mouse, one-way ANOVA with Tukey’s multiple comparisons test [F(3,8) = 29.20)]. (**D**) Cumulative frequency distribution plot of RNA foci volume. The yellow shaded area represents the cut-off for a cluster, which is defined to be at least 0.6 µm^3^. (**E**) Heatmap of the number of nuclear and cytoplasmic *Hs HTT1a* mRNA foci detected per individual cell by FISH. Each column adds up to 1. (**F**) Venn diagram depicting the co-localization of *Hs HTT* and *Hs HTT1a* mRNA analysed separately as foci versus clusters. (**G**) Scatter plot showing the volume of individual mRNA foci or clusters (see Methods for how volume was calculated). Each point represents the volume of individual mRNA foci and thick line represents the mean (Kruskal–Wallis one-way ANOVA with Dunn’s multiple comparisons test). (**H**) Same as (**B**) using different RNAscope probes. (**I**) Nuclear fraction of *Hs HTT1a* and *Hs HTT i66* mRNA is in the striatum and cortex. Each point represents a cell (*n* = ∼300 cells pooled from three mice per brain region, Kruskal–Wallis one-way ANOVA with Dunn’s multiple comparisons test). (**J**) Same as (**E**), but looking at *Hs HTT i66*. (**K**) Same as (**F**), but looking at the co-localization of *Hs HTT* clusters and *Hs HTT i66* foci. For all panels, ns = not significant, *P < 0.05, **P < 0.01, ***P < 0.001, ****P < 0.0001.

To interrogate what other components may be involved in *Hs HTT* clusters, we looked at the aberrantly spliced *HTT* exon 1-intron 1 fragment (*HTT1a*).^13^*HTT1a* is produced through repeat-driven interference with normal splicing at the exon 1-intron 1 junction, resulting in the formation of a ∼7.3-kb mRNA variant through the use of the cryptic polyA site in intron 1^13^ (Fig. 4A). *HTT1a* RNA and the translated protein fragment are detectable in post-mortem juvenile HD brain, and to a lesser extent, adult HD brain. However, detection has been challenging due to massive cell death in affected areas at the time of death.^14^

We evaluated and quantified nuclear and cytoplasmic foci per cell, foci/cluster volume, and cumulative frequency distribution of *Hs HTT1a,* full-length *HTT* mRNA and *HTT intron 66 (Hs HTT i66),* an intron in the pre-mRNA that marks the transcriptional site. To ensure specificity of detection, probes were designed to either the 5′ or 3′ end of *HTT1a* intron 1 (Fig. 4A). Complete co-localization between these two probes was observed (Fig. 4B, top row), confirming specificity and the presence of the predicted ∼7.3-kb *HTT1a* mRNA variant.^13^

Most *Hs HTT1a* signal was present in a single nuclear cluster, with 75% striatal neurons and 67% cortical neurons containing such clusters (Fig. 4C and D). Half of the cells, in addition, had at least one small cytoplasmic foci (Fig. 4E**)**. Qualitative and quantitative analysis showed almost complete co-localization of *Hs HTT* and *HTT1a* in clusters (>90%) but not in foci (<10%), indicating that the mRNA nuclear clusters represent a single molecular entity (Fig. 4B, bottom row, Fig. 4F). *Hs HTT1a* cluster volumes were similar in the striatum and cortex, reaching up to ∼3.8 µm^3^ (Fig. 4G).

To determine whether nuclear clustering was specific to the *HTT* mRNA variant containing the intron 1, we used a probe for *Hs HTT i66* (Fig. 4H), which marks unsliced, *HTT* pre-mRNA. *Hs HTT i66* did not form clusters (Fig. 4C, D, G and H) and, as expected, was only detectable as individual foci in the nucleus (Fig. 4H–J). Thus, co-clustering and nuclear export appear to be specific to *Hs HTT1a* and are likely caused by a repeat expansion.

*Hs HTT i66* foci mark active *Hs HTT* transcription sites.^42,43^ We observed 0–4 *Hs HTT i66* foci per cell (Fig. 4J), consistent with YAC128 mice carrying four copies of the *Hs HTT* transgene integrated at the same genomic loci.^42,43^ Interestingly, 97% of *Hs HTT/HTT1a* clusters co-localized with *Hs HTT i66* foci (Fig. 4K), indicating that clusters are forming near the chromosomal locations of the transgene. The proximity of Hs HTT transgene transcription might increase the cluster’s size by multivalent CG base-pairing interactions.^25^ These results support cis-formation of the *HTT/HTT1a* cluster.

To gain insight into the potential role of transcription and splicing in *HTT*/*HTT1a* mRNA cluster formation, we investigated whether clusters co-localized with established markers of splicing and high-efficiency transcription sites. Using a combination of FISH and immunofluorescence, we evaluated co-localization of Hs *HTT* mRNA clusters with splicing speckle marker SC35 protein, which marks transcriptionally active interchromatin structures^44,45^ and is implicated in assisting the formation of RNA clusters in other repeat expansion disorders. However, we observed no co-localization (Supplementary Fig. 8, bottom row). Previous studies reported co-localization of expanded CAG RNAs and SC35 used *in vitro* overexpression systems, which are likely responsible for the observed differences.^25,26^

The scaffolding long non-coding RNA *Mm Neat1* is also implicated in repeat-associated disorders.^46–48^ However, we observed no co-localization with *HTT1a/HTT* clusters in wild-type or YAC128 mice (Supplementary Fig. 8). In fact, *Mm Neat1* expression was bimodal: high levels in non-neuronal cells and low levels in neuronal cells. This result is consistent with a recent study reporting preferential expression of *Neat1* in astrocytes.^49^ Such bimodal distribution would prevent *Mm Neat1* from overlapping with neuron-selective clusters. We also saw no co-localization between clusters and nucleolin (not shown).

Collectively, these data suggest that *Hs HTT/HTT1a* mRNA clusters may not be associated with sites of efficient transcriptional or splicing activity but are spatially localized at a transcriptional locus.

### Nuclear *HTT1a* clusters and cytoplasmic foci are present in HD patient brain

**Figure 5:**
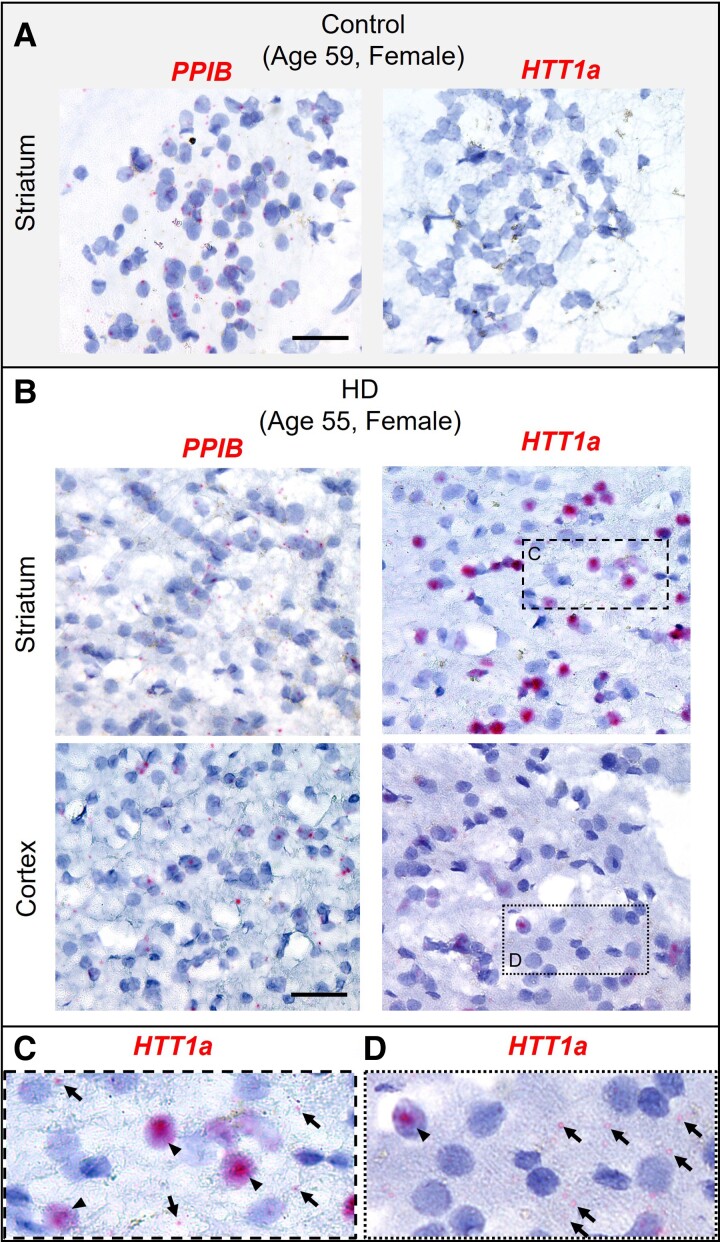
***Hs* HTT1a forms clusters in post-mortem HD brain and are detectable in the cytoplasm as foci** Chromogenic RNAscope assay was performed in healthy control and HD post-mortem human brains and counterstained with hematoxylin. (**A**) *PPIB* and *HTT1a* mRNA in post-mortem control striatum. Scale bar, 20 µm. (**B**) *PPIB* and *HTT1a* mRNA in post-mortem HD striatum (*top*) and cortex (*bottom*). Scale bar, 20 µm. (**C**, **D**) Insets of boxed regions in panel (**B**) showing *HTT1a* mRNA in the striatum (**C**) and cortex (**D**). Arrowheads indicate *HTT1a* clusters, and arrows indicate cytoplasmic *HTT1a* foci.

To investigate whether *HTT1a* clusters are present in human brains, we assayed post-mortem striatum from an HD patient (age 55, female, adult-onset) and an age-matched control (age 59, female, accidental death). We used a chromogenic version of RNAscope because high levels of autofluorescence caused by lipofuscin, protein accumulating in aging human brains, interfered with the use of the fluorescent assay.^50^*PPIB* (housekeeping gene) was detectable as discrete foci and did not form clusters in either control or HD brain (Fig. 5). In control brain, *HTT1a* mRNA was not detected (Fig. 5A). In the HD brain, cells with intense nuclear *HTT1a* clusters were easily detectable (visually occupying significant part of nuclear space) (Fig. 5B–D). The fraction of cells containing *HTT1a* clusters highly varied between sub-regions of the brain and different fields of view (representative images are shown in Supplementary Fig. 9), indicating a high degree of mosaicism. The presence of cells with distinct *HTT1a* nuclear clusters was much more prominent in the striatum compared to the cortex, consistent with HD clinically affecting the striatum first. Thus, nuclear *HTT1a* clustering, a phenomenon characterized in partially humanized mouse models, is observable in human brain tissue as well.

### ASOs silence *Mm Htt* and *Hs HTT* mRNA foci but not nuclear mRNA clusters

There is currently no cure for HD progression, but advances in oligonucleotide therapeutics have put effective treatments within reach.^51–57^ ASOs that block expression of *HTT* mRNA by inducing nuclear RNA degradation through ribonuclease H1 are in the clinic.^56^

**Figure 6:**
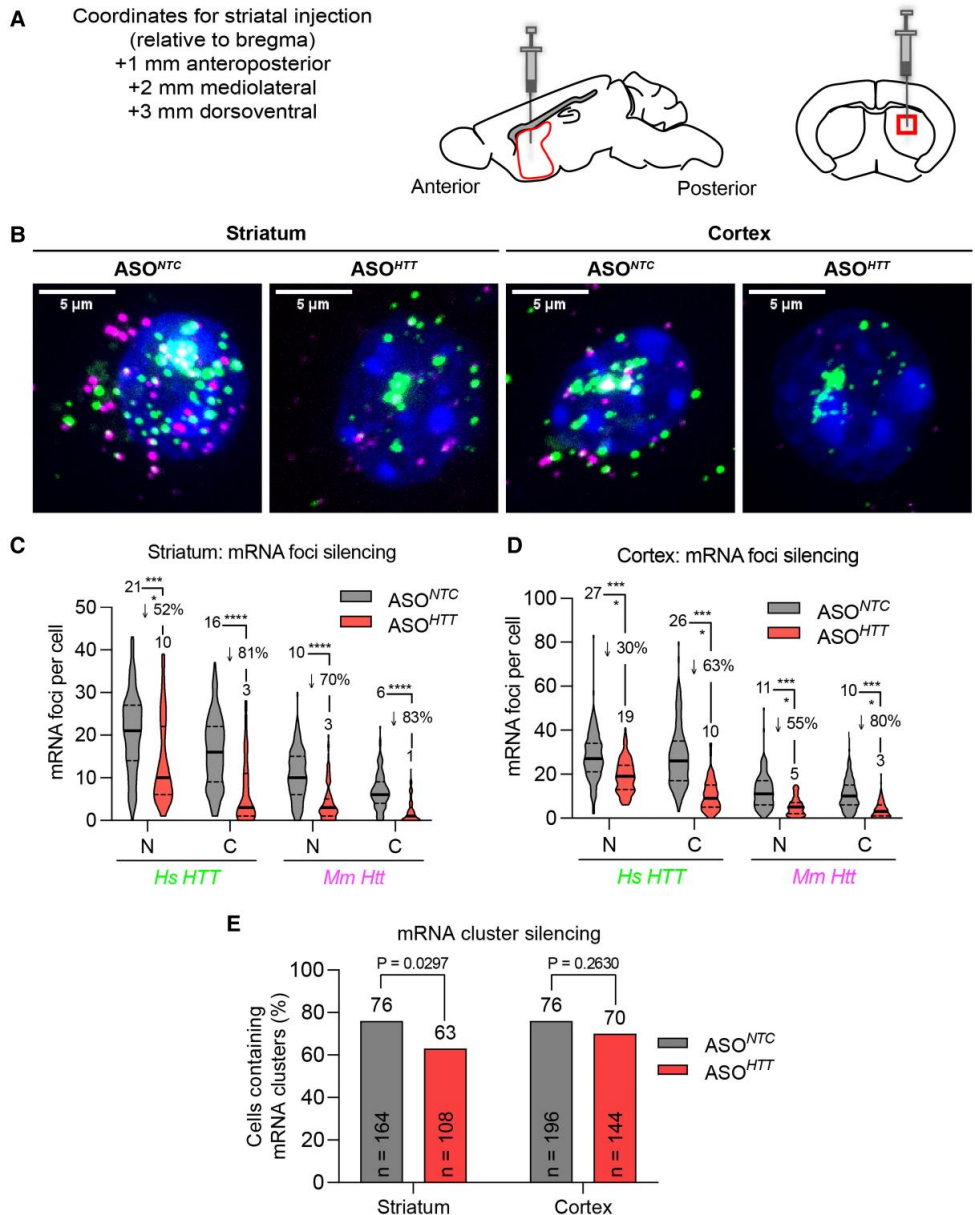
**ASOs efficiently silence wild-type Mm Htt and Hs HTT mRNA foci but not nuclear clusters.** (**A**) ASO*^NTC^* and ASO*^HTT^* (40 µg in 2 µl; *n* = 3 animals per group) were administered by unilateral intra-striatal bolus microinjection in 3-month-old YAC128 mice and euthanized 3 weeks later for analysis. Schematic diagram of sagittal and coronal sections through the mouse striatum at the site of injection is shown. The striatal region selected to acquire the images (box) is indicated. (**B**) FISH detection of *Mm Htt* and *Hs HTT* mRNAs in striatum (left) and cortex (right). Nuclei labelled with Hoechst. Representative images are maximum Z-projections through the nuclear region spaced 0.5 µm apart. Scale bar, 5 µm. (**C**, **D**) Quantification of *Hs HTT* and *Mm Htt* mRNA foci silencing in striatum (**C**) and cortex (**D**). N = nucleus, C = cytoplasm (*n* = 100–200 cells analysed per brain region per group pooled from three mice, *P < 0.05, **P < 0.01, ***P < 0.001, ****P < 0.0001, one-way ANOVA, Bonferroni’s multiple comparisons test). (**E**) Quantification of mRNA cluster silencing in striatum and cortex (*P* value calculated using Fisher’s exact test). ASO, antisense oligonucleotide; NTC, non-targeting control. See also Supplementary Table 2.

To evaluate the ability of HTT-targeting ASO to impact mRNA nuclear clusters, YAC128 mice were treated with ASOs targeting both *Mm Htt* (mRNA position 3168) and *Hs HTT* (mRNA position 3203).^33^ Three-month-old mice were injected with 40 µg ASO*^NTC^* (non-targeting control) or ASO*^HTT^* into the right striatum (*n* = 3 animals/group) (Fig. 6A). *Mm Htt* and *Hs HTT* RNA localization and clusters were evaluated by RNAscope 3 weeks after injection in 100–200 randomly selected cells per group (Fig. 6B). Consistent with our previous report in wild-type animals,^28^ ASO*^HTT^* efficiently silenced individual cytoplasmic *Mm Htt* and *Hs HTT* mRNA foci (∼80% in striatum; ∼60–80% in cortex, *P* < 0.0001 compared to ASO*^NTC^*). Reduction of nuclear *Hs HTT* foci was less pronounced, with 52 and 30% reduction in the striatum (Fig. 6C) and cortex (Fig. 6D), respectively (*P* < 0.0001 compared to ASO*^NTC^*). Furthermore, while ASO*^HTT^* potently silences individual mRNA foci, it had a minimal effect on presence of *Hs HTT* clusters in the majority of cells (*n* > 108 cells per group) (Fig. 6E; Supplementary Fig. 7). In striatum, 76% of cells were cluster-positive in the ASO*^NTC^* group compared to 63% in the ASO*^HTT^* group (*P* = 0.0297, Fisher’s exact test). In the cortex, 76% of cells were cluster positive in ASO*^NTC^* mice compared to 70% in ASO*^HTT^* mice (*P* = 0.2630, Fisher’s exact test). The reduced level of silencing in cortex compared to striatum is likely due to a lower level of ASO distribution away from the injection site (striatum). While the number of *Hs HTT* clusters in the striatum was significantly decreased in the ASO-treated group (Fig. 5E), it was barely pronounced compared to the level of *Hs HTT* foci silencing (Fig. 5C, *P* < 0.0001). These data suggest that nuclear *Hs HTT* clusters are resistant to ASO-mediated silencing at the time point tested.

## Discussion

High-resolution single-cell techniques are essential to uncovering molecular variations in disease that would otherwise be missed with gross tissue analysis. Using single-cell analysis, we investigated the effect of repeat expansion on intracellular localization of *huntingtin* mRNA *in vivo*. In HD mouse models, we find increased nuclear retention of *mHTT* mRNA foci and widespread presence of nuclear clusters (multiple mRNAs) in the majority of striatal and cortical neurons. The formation of clusters is dependent on the presence of expanded repeats, is likely caused by co-expression of the mis-spliced mRNA variant, *HTT1a*, and is observed as early as 1 month of age. Thus, nuclear *mHTT* clusters represent an early, robust molecular signature of HD, preceding any major transcriptomic, biochemical, or behavioural changes. Furthermore, nuclear *HTT1a* mRNA clusters are detectable in HD but not in normal patient brain slices and are resistant to ASO modulation, which are relevant factors for the design of future HD clinical interventions.

Our findings add HD to the list of repeat-associated neurodegenerative disorders in which the formation of nuclear mRNA clusters is a hallmark. The unstable CTG repeat expansion in the *DMPK* gene causes myotonic dystrophy 1,^58–61^ while a CCTG repeat expansion in the *CNBP* gene causes myotonic dystrophy 2.^62,63^ In both diseases, mutant CCUG-expanded RNAs sequester the muscleblind-like family of splicing factors, resulting in inappropriate splicing of various genes.^64–66^ In ALS, the presence of nuclear RNA foci containing GGGGCC-repeats correlates with inflammation in an interferon-induced manner by activating the protein kinase R stress pathway to enhance toxic peptide-dependent neurodegeneration.^67,68^

**Figure 7:**
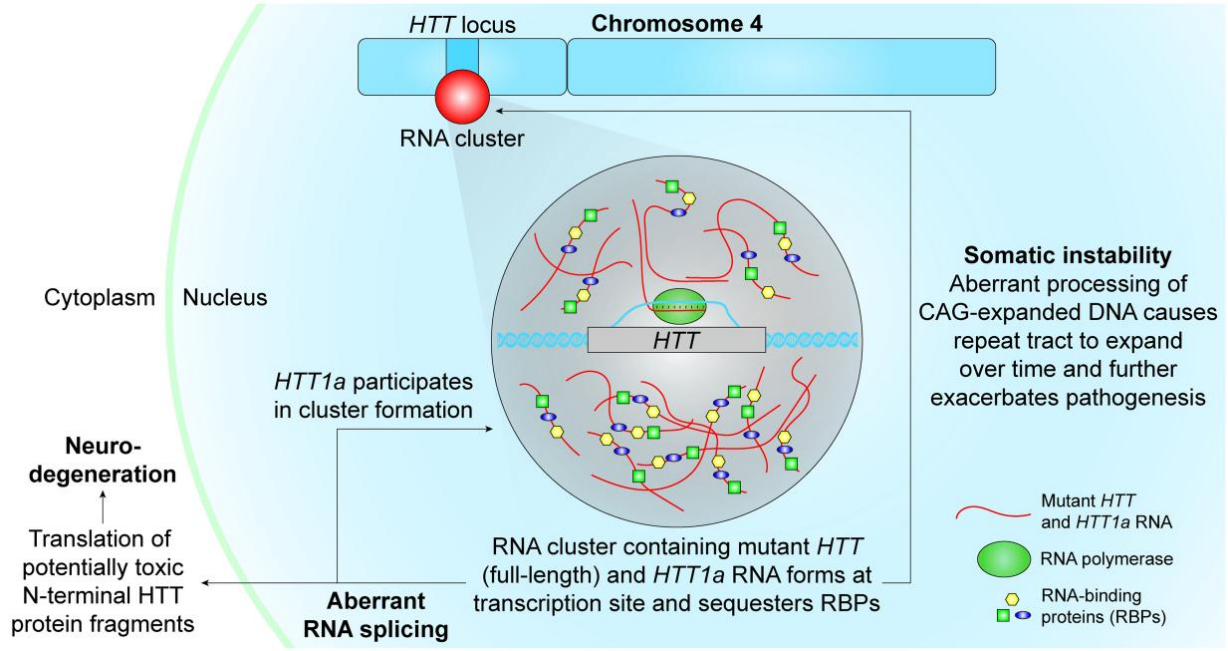
**Proposed model of mutant HTT RNA cluster formation, pathology, and pathogenesis.** RNA clusters containing mutant *HTT* RNA nucleate at active transcription sites. These repeat-expanded RNAs sequester RBPs, removing them from the available cellular pool, and thus, disrupting downstream RNA processing such as splicing. *HTT* itself is aberrantly spliced to produce *HTT1a*, which also participates in cluster formation. Globally disrupted splicing can result in the translation of altered protein isoforms and lead to neurotoxicity. This entire process is exacerbated by somatic instability, which acts as a positive feedback loop and expands the CAG repeat tract in the *HTT* gene over time and further increases the rate of *HTT1a* production.

Another HD-shared feature observed in repeat-associated neurodegenerative diseases is aberrant RNA processing, which has been reported in post-mortem HD brains.^69,70^ Such events include mis-splicing of *HTT* mRNA to produce *HTT1a*.^13^ Nuclear *HTT1a* mRNA, in turn, contributes to nuclear mutant mRNA clustering, potentially ramping up a self-regulation loop. In HD mice, the single *mHTT* mRNA cluster is likely formed by actively transcribed *mHTT* RNA at the genomic location on the chromosome. In this scenario, nascent RNA proximity would favour multivalent GC base-pairing, resulting in RNA cluster nucleation.^25^*mHTT* RNA clustering at active transcription sites (Fig. 7) might sequester RNA-binding proteins (RBPs), further disrupting downstream RNA processing such as splicing.^25^

Based on this study, we speculate that the levels of *HTT1a* mRNA expression are proportional to the rate of mis-splicing (i.e. production of *HTT1a* mRNA) and are higher in the context of longer and homogeneous (CAG versus CAG/CAA) repeats. We also observe that at an older age in YAC128, there is a downward trend toward in the amount of full-length nuclear *HTT* mRNA per cell (Supplementary Fig. 2D, mean = 23 nuclear foci at 3 months old versus 19 nuclear foci per cell at 8 months old, *P* < 0.0001). This change might be due to selective degeneration of neurons with a high degree of intranuclear clusters.

Although *mHTT* RNA clusters might be involved in disease progression by sequestering cellular factors necessary for healthy homeostasis, it is also possible that the formation of the nuclear mutant mRNA clusters may protect against pathogenesis by sequestering *HTT1a* and *mHTT* mRNA in the nucleus, thus preventing translation of toxic protein or N-terminal HTT fragments.^13,71^ This situation would be congruent to the formation of intranuclear HTT protein aggregates,^12,72^ where a role in pathogenesis^17,18^ and neuroprotection^73,74^ has been reported.

The presence of nuclear *HTT1a* clusters in HD patient brains highlights the potential clinical significance of *mHTT* clustering. Unlike HD mice—where each neuron contains the expanded repeat from birth, and cluster formation is observed in the majority of neurons—the frequency of *HTT1a* clusters in HD patient brains differed between sub-regions. Some regions displayed less than ∼1% of cells with clusters, while in other regions such as the striatum, clusters were more prevalent (> 5% cells). The mosaicism in cluster frequency in the HD human brain might be related to the somatic expansion of CAG repeats. Somatic repeat expansion, which is now considered a major modifier of HD progression,^75^ causes mosaic lengthening of CAG repeat tracts over time. This expanded CAG tract may sequester RBPs and result in aberrant splicing, thereby increasing *HTT1a* expression. At least ∼60 repeats are required for appreciable *HTT1a* expression.^15^ With the majority of adult-onset HD patients having around 40 repeats, the expression of *HTT1a* and clustering would likely be observed in neurons where the somatic expansion has pushed the number of CAG repeats above the ∼60 CAG threshold. Indeed, somatic repeat expansion is highly region- and tissue-specific, with the striatum being the most affected region in the brain. Indeed, this is the same trend observed with *HTT/HTT1a* mRNA nuclear clustering in HD models and patient samples. The relationship between somatic expansion and nuclear *HTT1a* RNA clusters in human brains warrants future detailed examination at the single-cell level.

HTT-lowering is accepted as a viable therapeutic paradigm for the treatment of HD.^52,53,76^ Recent technological advances allow highly-efficient modulation of *huntingtin* expression at the post-transcriptional level—i.e. ASOs,^77–79^ small interfering RNA (siRNA),^51,80,81^ and AAV-miRNA^82,83^—and at the transcriptional level—i.e. TALENs^84^ and small molecules.^85^ ASOs act in the nucleus and are usually highly effective in silencing localized nuclear transcripts from post-transcriptional modifications. Nuclear *mHTT* mRNA clusters were resistant to ASO-mediated silencing, which might be a result of a combination of enhanced stability and reduced accessibility.^86^ If *HTT* mRNA clusters are dynamic, an equilibrium between non-clustered and clustered mRNA may exist. A higher ASO dose or treatment period might have an effect on reducing nuclear mRNA clusters.

Recently released data from an ongoing clinical trial on an ASO targeting *HTT* showed potent modulation of mutant HTT protein in the CSF, but failed to meet primary clinical endpoints (ClinicalTrials.gov NCT03761849). At this point, the lack of significant clinical efficacy is not fully understood and the inability of ASOs to potently modulate mutant *HTT* mRNA clusters may a possible explanation. Therefore, if the inability of oligonucleotides to affect nuclear mRNA clustering is shown to be clinically significant, modalities that block *HTT* transcription (e.g. CRISPR/Cas9, TALENs) or directly target either the repeat tract or *HTT1a* isoform might be clinically advantageous.

At the time of this study, we used the B97-ΔN17 and YAC128 mouse models due to their popularity within the field and lack of data regarding mutant *HTT* mRNA subcellular localization. However, we do recognize the limitations of these models, such as a 17 amino acid deletion and interrupting CAA codons to stabilize the repeat length. Recently, a compelling new mouse model, called BAC-CAG, was recently reported from the Yang lab and has somatically unstable CAG repeats without CAA interruptions and a progressive disease phenotype that resembles HD patients.^87^ It would be very interesting to investigate the subcellular localization of *HTT* and *HTT1a* mRNA in these mice. Furthermore, it would be interesting to include the cerebellum in future studies, a region of the brain that is relatively spared compared to the striatum and cortex.

While preparing this manuscript, another recently published study from the Bates lab recapitulates the data in the present study, further solidifying the potential importance of RNA clusters in HD.^88^ Additional work is necessary to delineate and link the molecular mechanisms involved in *mHTT* mRNA clustering to HD pathology. However, this work provides clear evidence identifying mutant mRNA nuclear aggregation as one of the biomolecular signatures of HD, thus adding HD to a growing list of repeat-associated, neurodegenerative disorders with the demonstrated abnormalities in RNA processing and localization.

## Supplementary Material

fcac248_Supplementary_Data

## Abbreviations

AAV =: adeno-associated Virus
ALS =: amyotrophic lateral sclerosis
ASO =: antisense oligonucleotide
CRISPR/Cas9 =: clustered regularly interspaced short palindromic repeats/CRISPR-associated protein 9
CSF =: cerebrospinal fluid
DM1/2 =: myotonic dystrophy ½
FTD =: frontotemporal dementia
GWAS =: genome-wide association study
HD =: Huntington’s disease
miRNA =: microRNA
PBS =: phosphate-buffered saline
RAN =: repeat-associated non-AUG
RBP =: RNA-binding protein
RNAi =: RNA interference
SC35 =: serine/arginine-rich splicing factor SC35
SCA =: spinocerebellar ataxia
shRNA =: short hairpin RNA
siRNA =: small interfering RNA
SRSF1/2 =: serine and arginine-rich splicing factor ½
TALEN =: transcription activator-like effector nuclease
