# Supplementary material for: Mutant huntingtin messenger RNA forms neuronal nuclear clusters in rodent and human brains
Source: Brain Commun. 2022 Oct 13;4(6):fcac248. doi: 10.1093/braincomms/fcac248 (PMC9707646; doi:10.1093/braincomms/fcac248)
Supplement: fcac248_Supplementary_Data [file fcac248_supplementary_data.pdf]

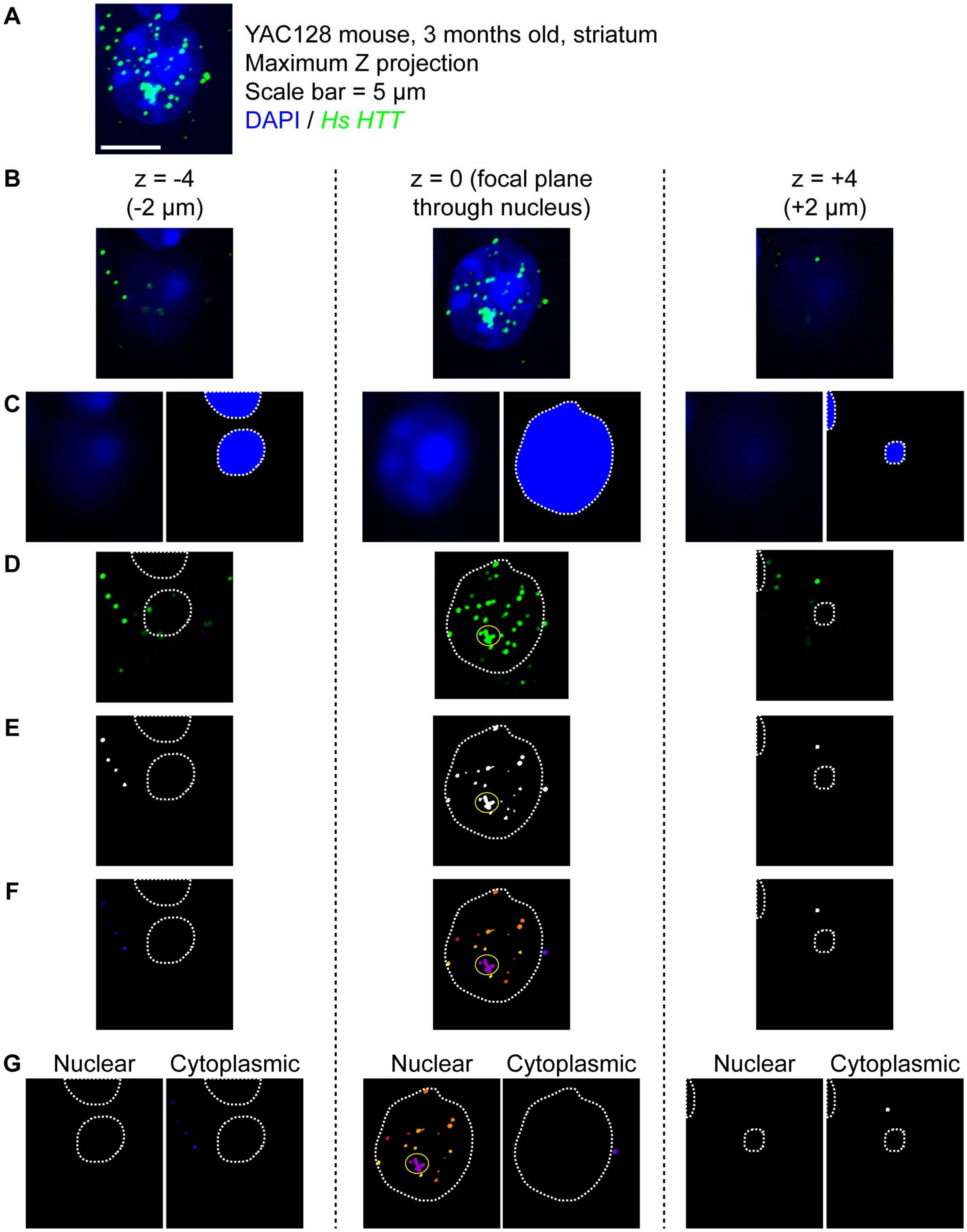
A
YAC128 mouse, 3 months old, striatum
Maximum Z projection
Scale bar = 5 μm
DAPI / Hs HTT
B
z = -4 (-2 μm)
z = 0 (focal plane through nucleus)
z = +4 (+2 μm)
C
D
E
F
G
Nuclear
Cytoplasmic
Nuclear
Cytoplasmic
Nuclear
Cytoplasmic

Supplementary Figure 1. Image processing for quantification of nuclear vs. cytoplasmic foci and volumes. Related to Figure 1.

(A) Maximum Z projection of a raw image stack (see Methods for image acquisition details). Image analysis was performed in 3D. For simplicity, only one RNAscope probe is shown (*Hs HTT*, green), but all RNAscope channels were processed identically. (**B**) Three individual planes of the image stack shown side-by-side as a comparison. (**C**) (Left) DAPI channel convolved with a Gaussian blur (σ = 10 pixels) and then (right) thresholded using the "Default" method in ImageJ v1.53c (stack histogram enabled). The resulting nucleus is outlined with a white dashed line and overlayed in all subsequent steps. (**D**) Difference of Gaussians filter (σ_1 = 2 pixels, σ_2 = 4 pixels) on the green RNAscope channel in (B). Yellow circle is outlining a cluster. (**E**) Thresholded image of (D) using the minimum intensity value calculated from either the Otsu threshold or a manual threshold (250 intensity). (**F**) 3D Objects Counter of (E) (minimum size = 25 voxels) with different colors representing discrete, contiguous objects that were detected. (**G**) Overlay of (F) with the nuclear mask in (**C**) to determine nuclear or cytoplasmic localization of RNAscope foci.

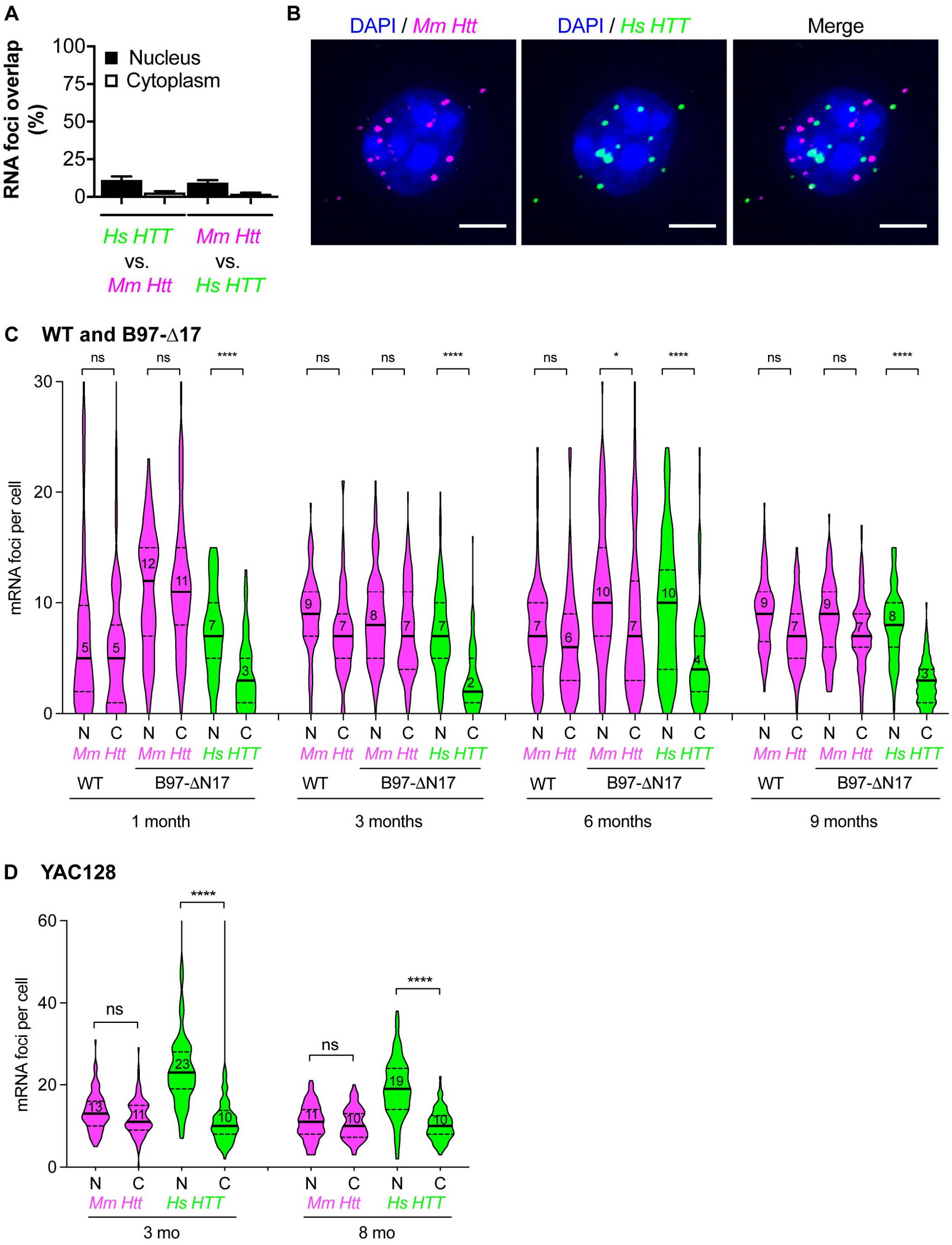

A
RNA foci overlap (%)
100
75
50
25
0
Nucleus
Cytoplasm
Hs HTT vs. Mm Htt
Mm Htt vs. Hs HTT
B
DAPI / Mm Htt
DAPI / Hs HTT
Merge
C
WT and B97-Δ17
mRNA foci per cell
ns
*
****
N
C
Mm Htt
Hs HTT
WT
B97-ΔN17
1 month
3 months
6 months
9 months
D
YAC128
3 mo
8 mo

Supplementary Figure 2. Validation of *Mm Htt and Hs HTT* mRNAs detection by multiplexed dual-color FISH labeling. Related to Figure 1, 2, and 3.

(**A**) Co-localization of *Hs HTT* (green) and *Mm Htt* (magenta) RNA in the nucleus (black bars) and cytoplasm (white bars) in B97-ΔN17 striatum. RNA foci overlap was calculated between different probe sets using ImageJ (n = 60 images pooled from three mice, 20 images per mouse; mean ± SEM). Minimal co-localization was observed. (**B**) Representative FISH image used for quantitative analysis in panel A. Nuclei labeled with Hoechst (blue). Scale bar, 5 µm. (**C**-**D**) Scatter plots representing the absolute quantification of *Mm Htt* (magenta) and *Hs HTT* (green) mRNA foci in the nucleus and the cytoplasm in (**C**) wild-type (WT), B97-ΔN17 mice and (**D**) YAC128 mice. For panels C and D, n = 3 mice per group and at least 30 images were acquired per mouse and pooled for quantitative analysis. ns = not significant, * $P < 0.05$, ** $P< 0.01$, *** $P < 0.001$, **** $P < 0.0001$, one-way ANOVA with Dunn's multiple comparisons test ($F(23, 2087) = 22.31$).

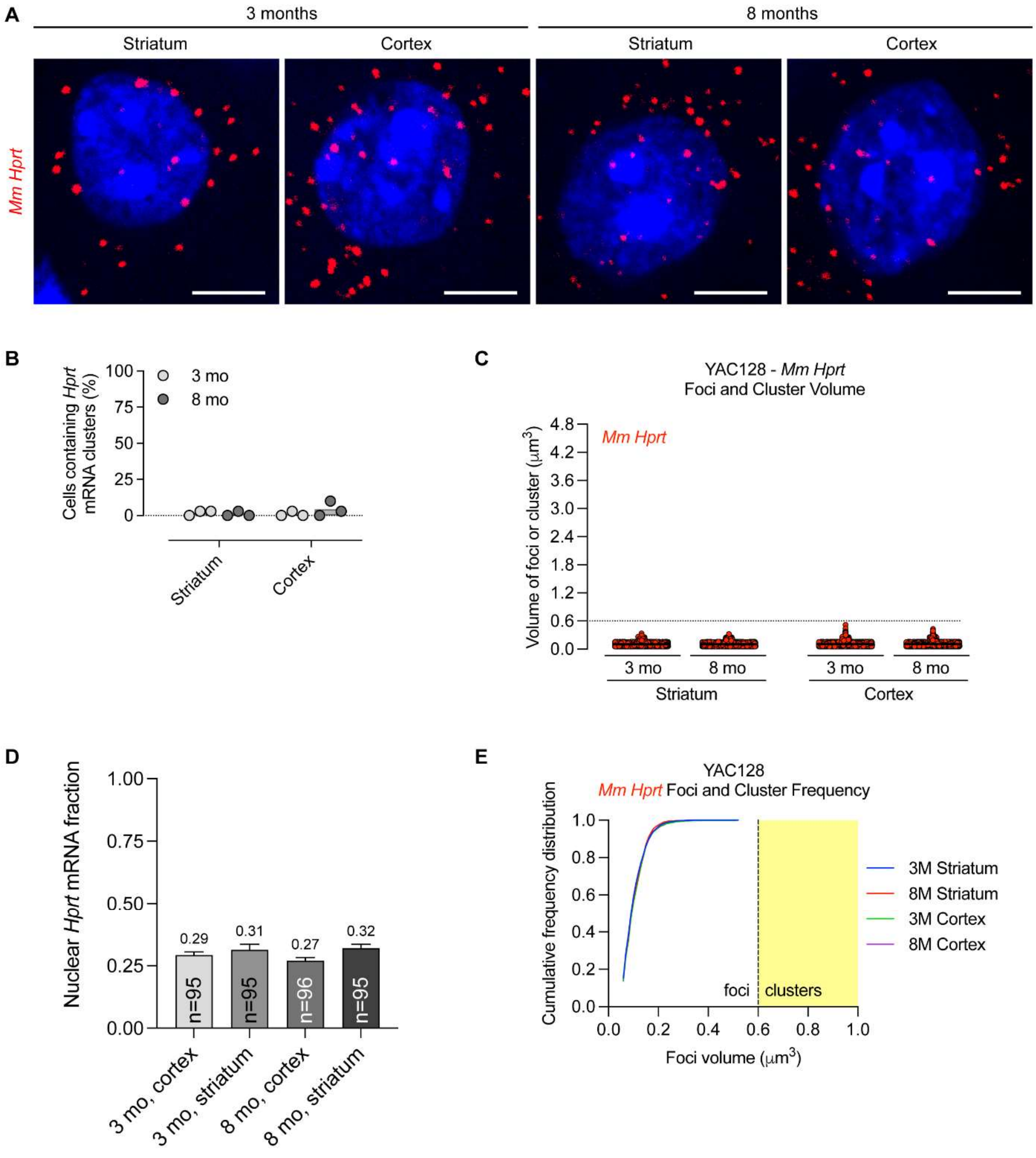

A
3 months
8 months
Striatum
Cortex
Striatum
Cortex
Mm Hprt
B
Cells containing Hprt mRNA clusters (%)
100
75
50
25
0
3 mo
8 mo
Striatum
Cortex
C
YAC128 - Mm Hprt
Foci and Cluster Volume
Mm Hprt
Volume of foci or cluster (μm3)
4.8
4.2
3.6
3.0
2.4
1.8
1.2
0.6
0.0
3 mo
8 mo
3 mo
8 mo
Striatum
Cortex
D
Nuclear Hprt mRNA fraction
1.00
0.75
0.50
0.25
0.00
0.29
0.31
0.27
0.32
n=95
n=95
n=96
n=95
3 mo, cortex
3 mo, striatum
8 mo, cortex
8 mo, striatum
E
YAC128
Mm Hprt Foci and Cluster Frequency
Cumulative frequency distribution
1.0
0.8
0.6
0.4
0.2
0.0
foci
clusters
0.0
0.2
0.4
0.6
0.8
1.0
Foci volume (μm3)
3M Striatum
8M Striatum
3M Cortex
8M Cortex

Supplementary Figure 3. *Mm Hprt* mRNA is mostly cytoplasmic and does not form clusters in YAC128 striatum and cortex. Related to Figure 2.

(**A**) *Mm Hprt* (red) mRNA in YAC128 mouse striatum and cortex detected by FISH. Nuclei labeled with Hoechst (blue). Representative images of maximum Z-projections. Scale bar, 5 µm. (**B**) Percentage of cells containing *Mm Hprt* mRNA clusters. Each point represents a mouse (n = ~100 cells pooled from 3 mice). (**C**) Scatter plots representing the volume (µm^3) of individual foci at 3 and 8 months in the striatum and cortex. Each dot represents an individual foci (n = ~100 cells pooled from 3 mice). (**D**) Nuclear percentage of *Mm Hprt* mRNA foci (mean $\pm$ SD, n =3 biological replicates). (**E**) Cumulative frequency distribution plot of RNA foci volume. Yellow shaded area represents the cut-off for a cluster, which is defined to be at least 0.6 µm^3. M = months old.

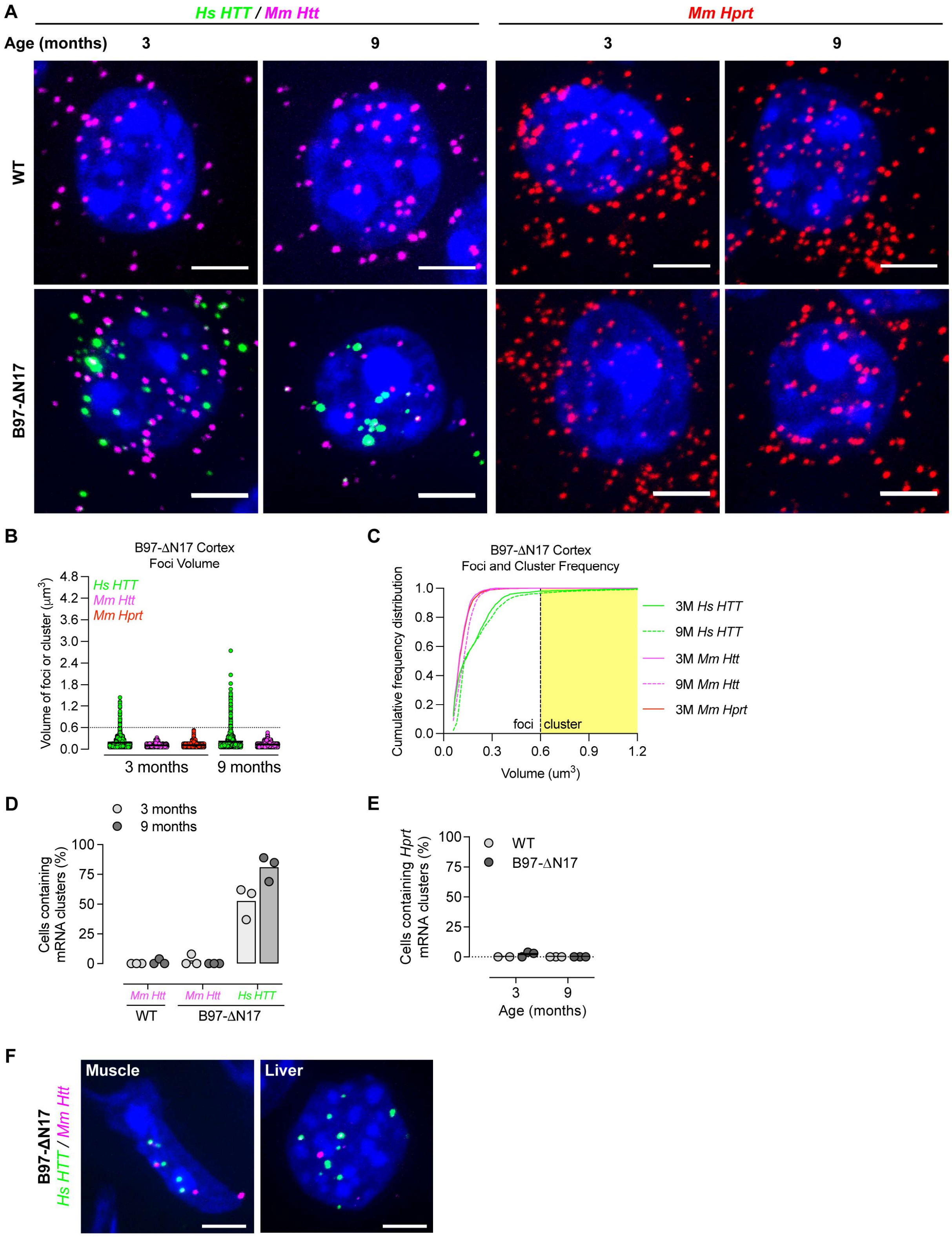
A
Hs HTT / Mm Htt
Mm Hprt
Age (months) 3 9 3 9
WT
B97-ΔN17
B
B97-ΔN17 Cortex
Foci Volume
Hs HTT
Mm Htt
Mm Hprt
Volume of foci or cluster (μm3)
3 months
9 months
C
B97-ΔN17 Cortex
Foci and Cluster Frequency
Cumulative frequency distribution
foci
cluster
Volume (um3)
3M Hs HTT
9M Hs HTT
3M Mm Htt
9M Mm Htt
3M Mm Hprt
D
3 months
9 months
Cells containing mRNA clusters (%)
Mm Htt
Mm Htt
Hs HTT
WT
B97-ΔN17
E
Cells containing Hprt mRNA clusters (%)
WT
B97-ΔN17
Age (months)
F
Muscle
Liver
B97-ΔN17
Hs HTT / Mm Htt

Supplementary Figure 4. Repeat-expanded *HTT* mRNA forms nuclear clusters in B97-ΔN17 mouse cortex. Related to Figure 3.

(**A**) *Hs HTT* (green), *Mm Htt* (magenta) and *Mm Hprt* (red) mRNAs detected in wild-type (WT, upper panel) and B97-ΔN17 (lower panel) mouse cortex by FISH. Nuclei labeled with Hoechst (blue). Representative images of maximum Z-projections through the nucleus. Scale bar, 5 µm. (**B**) Scatter plot showing the volume of individual mRNA foci or cluster (see Methods for how volume was calculated). (**C**) Cumulative frequency distribution plot of RNA foci volume. Yellow shaded area represents the cut-off for a cluster, which is defined to be at least 0.6 μm^3. M = months old. Thick line represents the mean. (**D, E**) Percentage of cells containing *Mm Htt*, *Hs HTT* or *Mm Hprt* mRNA clusters in WT and B97-ΔN17 mouse cortex (n = ~100 cells per brain region pooled from 2-3 mice, each point represents a mouse). For all panels, ns = not significant, ** $P < 0.01$, *** $P < 0.001$, **** $P < 0.0001$, one-way ANOVA, Bonferroni's multiple comparisons test. (**F**) *Hs HTT* (green) and *Mm Htt* (magenta) mRNAs detected in B97-ΔN17 mouse muscle and liver by FISH. Nuclei labeled with Hoechst (blue). Representative images of maximum Z-projections. Scale bar, 5 µm.

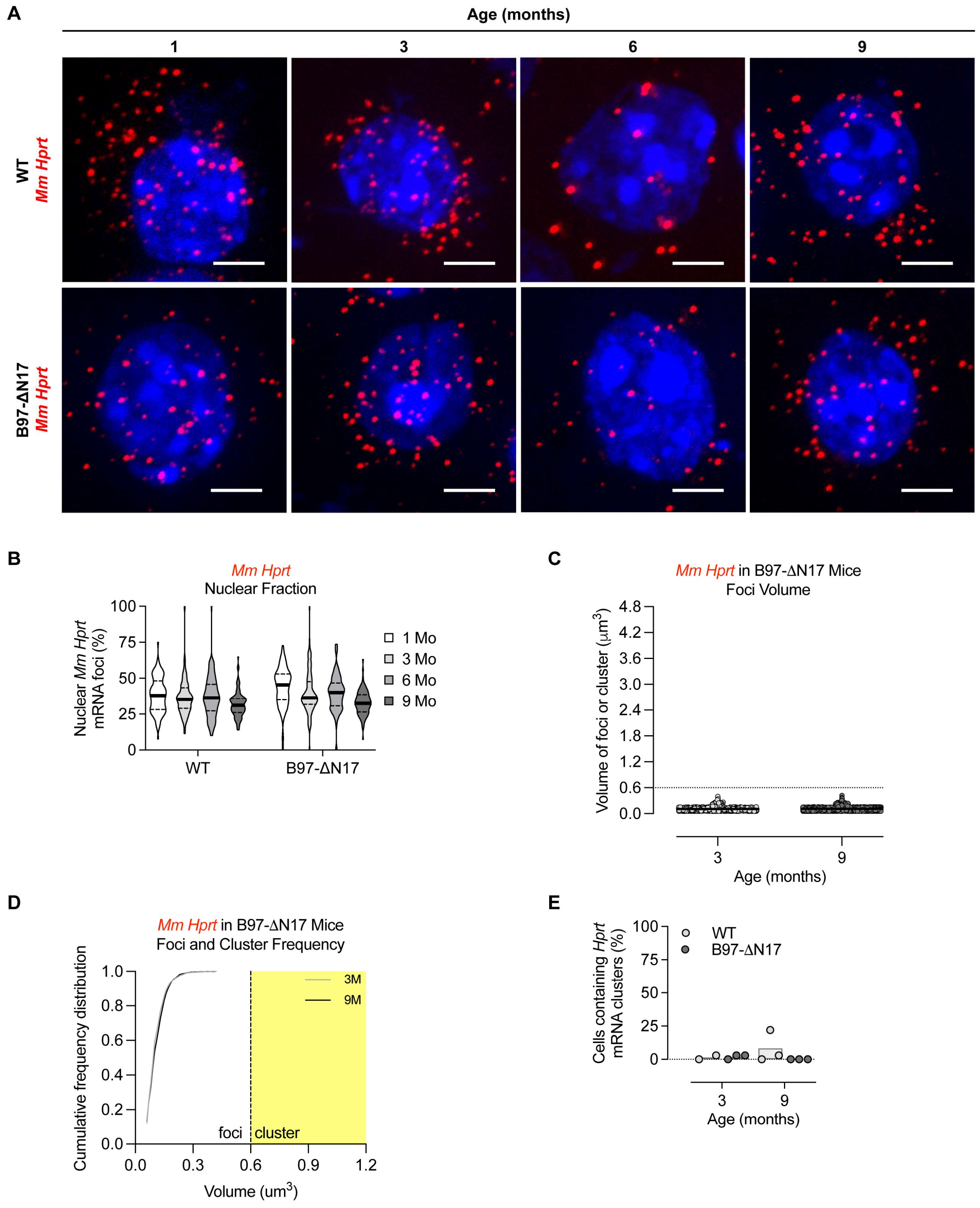
A
Age (months)
1
3
6
9
WT
Mm Hprt
B97-ΔN17
Mm Hprt
B
Mm Hprt
Nuclear Fraction
Nuclear Mm Hprt mRNA foci (%)
100
75
50
25
0
WT
B97-ΔN17
1 Mo
3 Mo
6 Mo
9 Mo
C
Mm Hprt in B97-ΔN17 Mice
Foci Volume
Volume of foci or cluster (μm3)
4.8
4.2
3.6
3.0
2.4
1.8
1.2
0.6
0.0
3
9
Age (months)
D
Mm Hprt in B97-ΔN17 Mice
Foci and Cluster Frequency
Cumulative frequency distribution
1.0
0.8
0.6
0.4
0.2
0.0
3M
9M
foci
cluster
0.0
0.3
0.6
0.9
1.2
Volume (um3)
E
Cells containing Hprt mRNA clusters (%)
100
75
50
25
0
WT
B97-ΔN17
3
9
Age (months)

Supplementary Figure 5. *Mm Hprt* mRNA is mostly cytoplasmic and does not form clusters in B97-ΔN17 mouse striatum. Related to Figure 3.

(**A**) *Mm Hprt* mRNA (red) detected in the striatum of wild-type (WT) and B97-ΔN17 mice by FISH. Nuclei labeled with Hoechst (blue). Representative images of maximum Z-projections. Scale bar, 5 μm. (**B**) Percentage of nuclear *Hprt* mRNA in wild-type (WT) and B97-ΔN17 mice at 1, 3, 6 and 9 months (n = ~100 cells pooled from 3 mice). (**C**) Scatter plot showing the volume of individual mRNA foci or cluster (see Methods for how volume was calculated). Thick line represents the mean. (**D**) Cumulative frequency distribution plot of RNA foci volume. Yellow shaded area represents the cut-off for a cluster, which is defined to be at least 0.6 μm^3. M = months old. (**E**) Percentage of cells containing *Mm Hprt* mRNA clusters in WT and B97-ΔN17 mouse striatum (n = ~100 cells per brain region pooled from 3 mice, each point represents a mouse). For all panels, ns = not significant, ** $P < 0.01$, *** $P < 0.001$, **** $P < 0.0001$, one-way ANOVA, Bonferroni's multiple comparisons test.

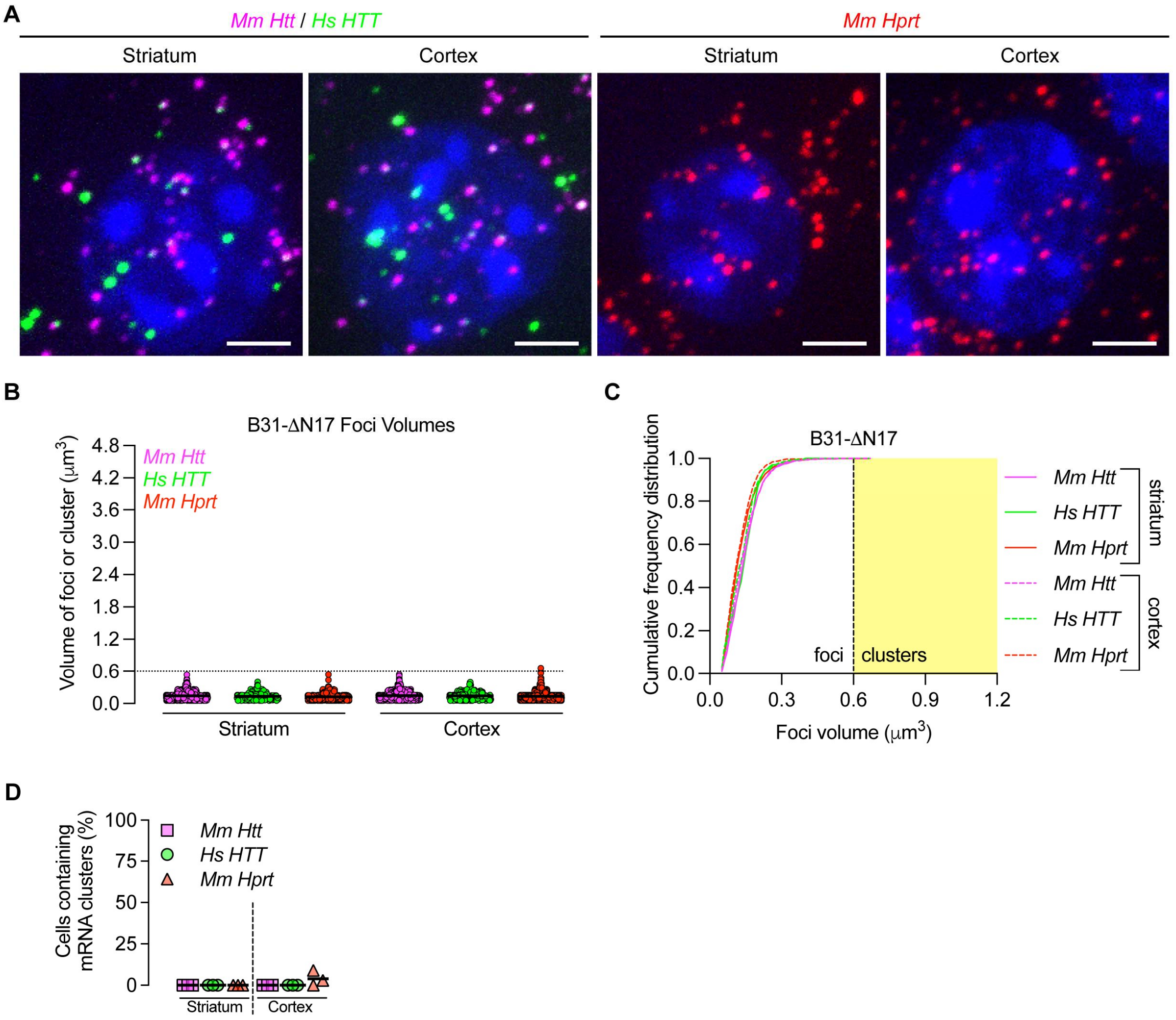

A
Mm Htt / Hs HTT
Mm Hprt
Striatum
Cortex
Striatum
Cortex
B
B31-ΔN17 Foci Volumes
Volume of foci or cluster (μm3)
4.8
4.2
3.6
3.0
2.4
1.8
1.2
0.6
0.0
Mm Htt
Hs HTT
Mm Hprt
Striatum
Cortex
C
B31-ΔN17
Cumulative frequency distribution
1.0
0.8
0.6
0.4
0.2
0.0
0.0
0.3
0.6
0.9
1.2
foci
clusters
Foci volume (μm3)
Mm Htt
Hs HTT
Mm Hprt
striatum
Mm Htt
Hs HTT
Mm Hprt
cortex
D
Cells containing mRNA clusters (%)
100
75
50
25
0
Mm Htt
Hs HTT
Mm Hprt
Striatum
Cortex

Supplementary Figure 6. Nuclear and cytoplasmic distribution of wild-type *Mm Htt* (7 CAG), *Hs HTT* (31 CAG) and *Mm Hprt* in B31-∆N17 (4 months old) mouse striatum and cortex. Related to Figure 3.

(A) FISH performed on B31-∆N17 (4 months old) mouse striatum and cortex. Nuclei stained with Hoechst (blue). Representative images are maximum Z projections through the nucleus region spaced 0.5 µm apart. Scale bar, 5 µm. (**B**) Scatter plot showing the volume of individual mRNA foci or cluster (see Methods for how volume was calculated). Thick line represents the mean. (**C**) Cumulative frequency distribution plot of RNA foci volume. Yellow shaded area represents the cut-off for a cluster, which is defined to be at least 0.6 $µm^3$. M = months old. **(D)** Percentage of cells containing mRNA clusters in B31-∆N17 mouse striatum and cortex (n = ~100 cells per brain region pooled from 3 mice, each point represents a mouse).

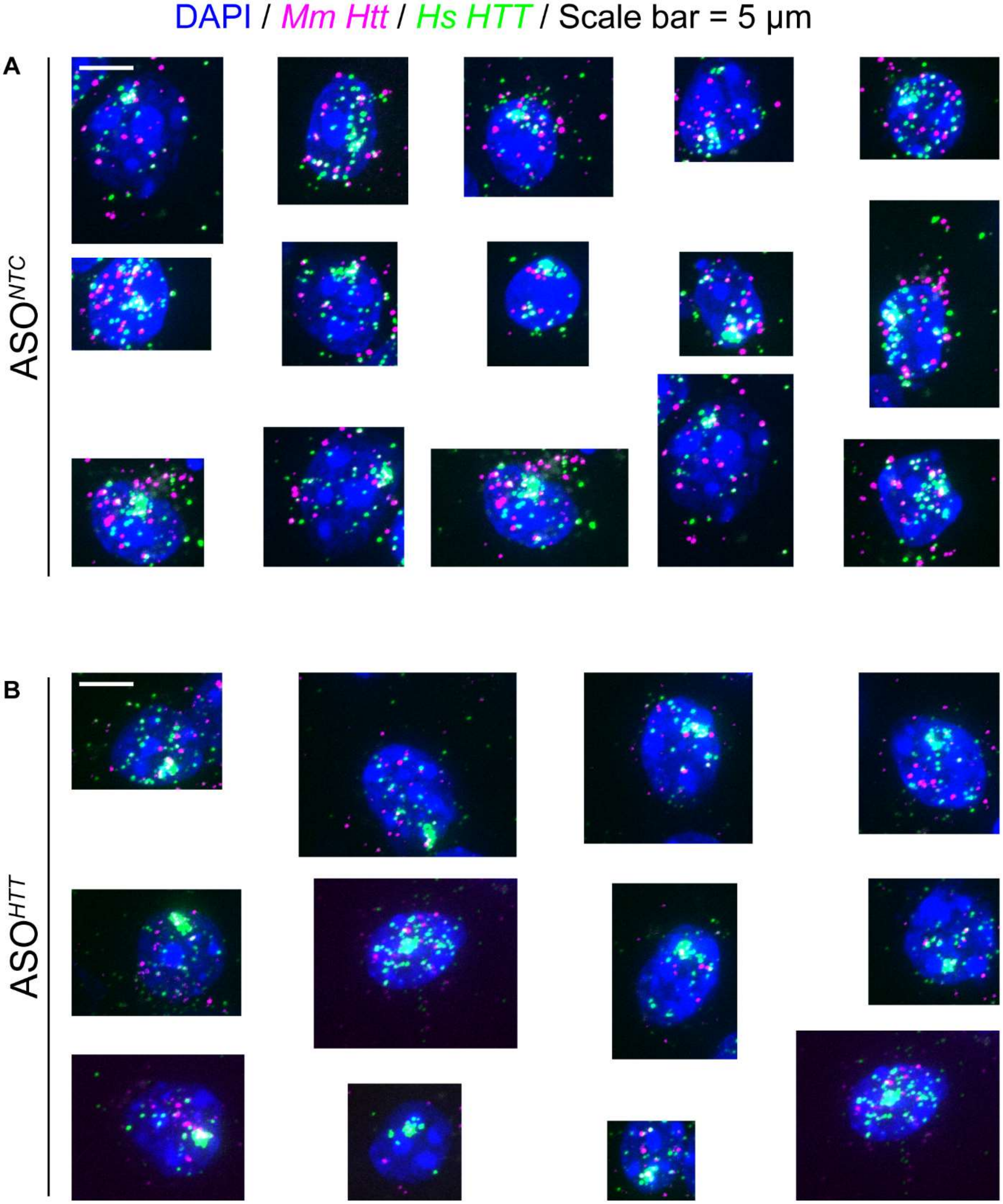
DAPI / *Mm Htt* / *Hs HTT* / Scale bar = 5 µm
A
ASO^{NTC}
B
ASO^{HTT}

Supplementary Figure 7: Mutant *Hs HTT,* but not wild-type *Mm Htt*, clusters are easily detectable in YAC128 (3 months old) striatum. Related to Figure 2, 3, and 6. Wild-type *Mm Htt* (magenta) and mutant *Hs HTT* (green) were detected in YAC128 (3 months old) mouse striatum. A random panel of images is shown for (**A**) ASO^{NTC}-treated mice and (**B**) ASO^{HTT}-treated mice. Representative images are maximum Z-projections through the nucleus spaced 0.5 µm apart. Nuclei stained with DAPI (blue). Scale bar, 5 µm. Nuclear clusters are resistant to ASO treatment.

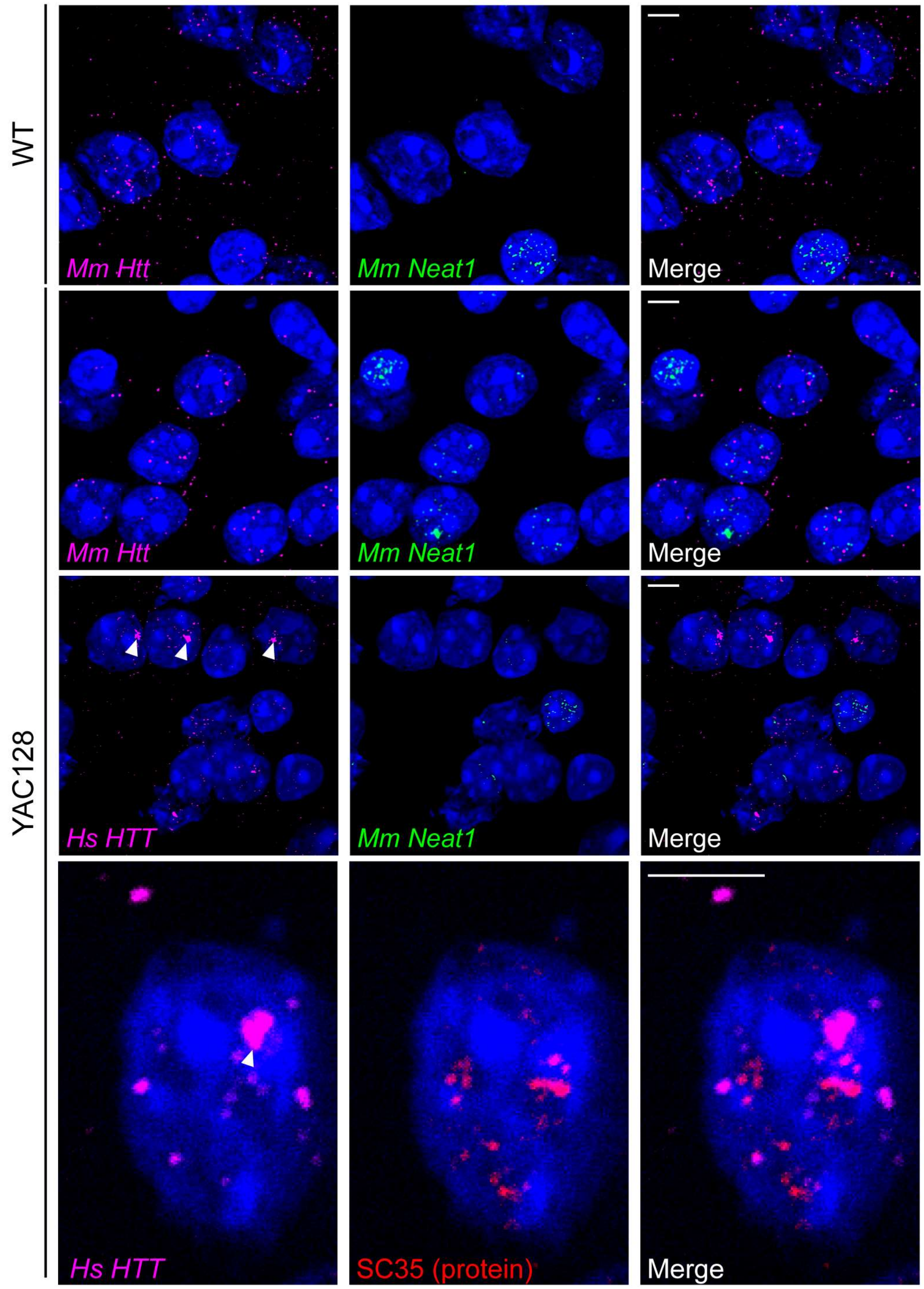
WT
YAC128
Mm Htt
Mm Neat1
Merge
Mm Htt
Mm Neat1
Merge
Hs HTT
Mm Neat1
Merge
Hs HTT
SC35 (protein)
Merge

Supplementary Figure 8. Nuclear *Hs HTT* mRNA clusters do not co-localize with the paraspeckle markers *Mm Neat1* (lncRNA) or SC-35 (protein). Related to Figure 4.

RNAscope was performed in WT and YAC128 mouse brain (3 months old). Cells highly expressing the paraspeckle marker *Mm Neat1* were not the same population of cells highly expressing *Hs HTT* containing clusters. Arrowheads indicate *Hs HTT* clusters. Scale bar, 5 µm.

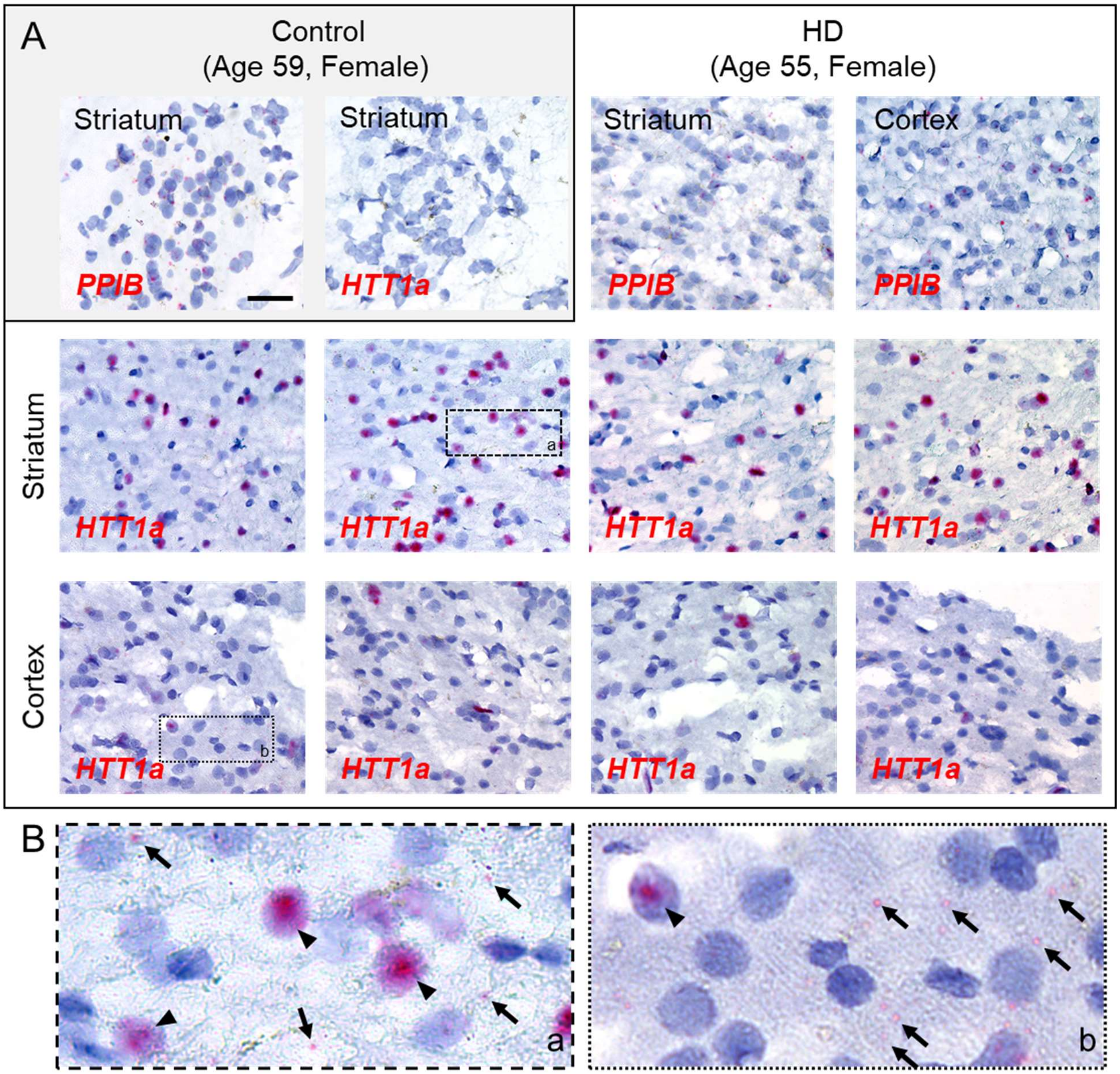
A
Control
(Age 59, Female)
HD
(Age 55, Female)
Striatum
Striatum
Striatum
Cortex
PPIB
HTT1a
PPIB
PPIB
Striatum
HTT1a
HTT1a
HTT1a
HTT1a
a
Cortex
HTT1a
HTT1a
HTT1a
HTT1a
b
B
a
b

Supplementary Figure 9. *Hs HTT1a* forms clusters in post-mortem HD brain and are detectable in the cytoplasm as foci

Chromogenic RNAscope assay was performed in healthy control and HD post-mortem human brains and counterstained with hematoxylin. (A) In the HD brain, four different fields view in the striatum and cortex using the *HTT1a* probe are shown to illustrate the variability in different subregions of the brain. Scale bars, 20 µm. (B) Insets of (a) HD striatum and (b) cortex from panel A. Arrowheads indicate and *HTT1a* clusters and arrows indicate cytoplasmic *HTT1a* foci.

SUPPLEMENTARY TABLES

HD Model	Model Type	Human region inserted	Repeat length	Age of Phenotype	Other notable features	Citation
BACHD-ΔN17-97Q	Transgene	Full CAG-expanded *HTT* transgene	97 mixed CAG/CAA	~6 months	BACHD-ΔN17-31Q (control); 51 nucleotide deletion in N-terminal	(Gray et al., 2008; Gu et al., 2015)
YAC128	Transgene	Full CAG-expanded *HTT* transgene	128 mostly pure CAG (82 uninterrupted)	~6 months		(Slow et al., 2003)

Supplementary Table 1: Summary of mouse models. Related to Figure 1.

Name	Sequence 5′-3′	Reference
ASO^{NTC}	+A#+A#+C#A#C#G#T#C#T#A#T#A#C#+G#+C	
ASO^{HTT}	#+A#+G#+C#A#T#C#C#A#A#A#T#G#T#+G#+A#G	(Didiot et al., 2018; Hung et al., 2015)

Supplementary Table 2. Hydrophobically modified ASO sequences and modifications. #, phosphorothioate; +, locked nucleic acid. Related to Figure 6 and Figure S7.

Species	Target RNA	ACDBio product #
Mouse	*Htt*	473001
Mouse	*Hprt*	312951
Mouse	*Neat1*	440351
Human	*HTT*	420231
Human	*HTT1a* (5′)	561431
Human	*HTT1a* (3′)	561441
Human	*HTT i66*	493761

Supplementary Table 3. Probe sets used for FISH experiments. Related to Figure 1.

SUPPLEMENTARY REFERENCES

Didiot, M.C., Ferguson, C.M., Ly, S., Coles, A.H., Smith, A.O., Bicknell, A.A., Hall, L.M., Sapp, E., Echeverria, D., Pai, A.A., *et al.* (2018). Nuclear Localization of Huntingtin mRNA Is Specific to Cells of Neuronal Origin. Cell reports *24*, 2553-2560.e2555.

Gray, M., Shirasaki, D.I., Cepeda, C., Andre, V.M., Wilburn, B., Lu, X.H., Tao, J., Yamazaki, I., Li, S.H., Sun, Y.E., *et al.* (2008). Full-length human mutant huntingtin with a stable polyglutamine repeat can elicit progressive and selective neuropathogenesis in BACHD mice. J Neurosci *28*, 6182-6195.

Gu, X., Cantle, J.P., Greiner, E.R., Lee, C.Y., Barth, A.M., Gao, F., Park, C.S., Zhang, Z., Sandoval-Miller, S., Zhang, R.L., *et al.* (2015). N17 Modifies mutant Huntingtin nuclear pathogenesis and severity of disease in HD BAC transgenic mice. Neuron *85*, 726-741.

Hung, G., Leeds, J., Bennett, C.F., and Freier, S.M. (2015). Compositions and their uses directed to huntingtin (United States: Isis Pharmaceuticals, Inc., CHDI Foundation Inc.).

Slow, E.J., van Raamsdonk, J., Rogers, D., Coleman, S.H., Graham, R.K., Deng, Y., Oh, R., Bissada, N., Hossain, S.M., Yang, Y.Z., *et al.* (2003). Selective striatal neuronal loss in a YAC128 mouse model of Huntington disease. Hum Mol Genet *12*, 1555-1567.
